# ISX-9 potentiates CaMKIIδ-mediated BMAL1 activation to enhance circadian amplitude

**DOI:** 10.1038/s42003-022-03725-x

**Published:** 2022-07-28

**Authors:** Huilin Li, Jiali Ou, Yaqun Li, Niannian Xu, Qing Li, Ping Wu, Chao Peng, Yun-Chi Tang, Hung-Chun Chang

**Affiliations:** 1 CAS Key Laboratory of Tissue Microenvironment and Tumor, Shanghai Institute of Nutrition and Health, University of Chinese Academy of Sciences, Chinese Academy of Sciences, Shanghai, 200031 China; 2 Institute of Neuroscience, State Key Laboratory of Neuroscience, CAS Key Laboratory of Primate Neurobiology, CAS Center for Excellence in Brain Science and Intelligence Technology, Chinese Academy of Sciences, Shanghai, 200031 China; 3 Shanghai Research Center for Brain Science and Brain-Inspired Intelligence, Shanghai, 201210 China; 4 National Facility for Protein Science in Shanghai, Zhangjiang Lab, Shanghai Advanced Research Institute, Chinese Academy of Science, Shanghai, 201210 China

**Keywords:** Circadian rhythms, Circadian rhythms and sleep

## Abstract

Circadian dysregulation associates with numerous diseases including metabolic dysfunction, sleep disorder, depression and aging. Given that declined circadian amplitude is a trait commonly found with compromised health, interventions that design in precluding circadian amplitude from dampening will aid to mitigate complex, circadian-related diseases. Here we identify a neurogenic small molecule ISX-9 that is able to support persistent and higher amplitude of circadian oscillations. ISX-9 improves diurnal metabolic rhythms in middle-aged mice. Moreover, the ISX-9-treated mice show better sleep homeostasis with increased delta power during the day time and higher locomotive activity in the dark period. ISX-9 augments CaMKIIδ expression and increases BMAL1 activity via eliciting CaMKIIδ-mediated phosphorylation on BMAL1 residues S513/S515/S516, accordingly composes a positive feedback effect on enhancing circadian amplitude. CaMKIIδ-targeting, and the use of ISX-9 may serve as decent choices for treating circadian-related disorders.

## Abstract (web summary)

Circadian modulators that exert amplitude-enhancing activity using highthroughput compound screening of small molecules identifies ISX-9, which is shown to augment CaMKIIδ expression, increasing BMAL1 activity via CaMKIIδ-dependent phosphorylation.

## Introduction

Circadian clock is an intrinsic timekeeper that coordinates multiple physiological activities in an orderly manner^1–3^. Environmental or genetic disruptions of circadian rhythms in model organisms have revealed a series of symptoms related to human diseases including cardiovascular dysfunction^4^, diabetic mellitus^2,5^, cancer^6^, sleep disorders^7–9^, depression^10–13^, neurodegenerative diseases^14^, and aging^15–18^. As circadian amplitude decay and phase mis-alignment are commonly associated with deteriorated health and aging^15,19,20^, interventions with supporting circadian amplitude and correcting phase mis-alignment will assist to ameliorate circadian-associated diseases including aging^21^. Several compounds have been identified to modulate circadian oscillation and strength^22–24^, and many of these small molecules target directly on circadian clock components such as REV-ERBs^25,26^, RORs^27,28^, and CRY1/2^29^, to tune the circadian core loops transcriptionally or post-translationally. Agonists for REV-ERBs such as GSK4112 and the derivatives SR9009/SR9011 showed efficacies with increased energy expenditure hence preventing obesity from high-fat diet (HFD) feeding^25^. SR9009/SR9011 were also demonstrated to act as anticancer agents^30^, suggesting that the stimulating of REV-ERBs is a decent strategy to combat metabolic dysregulation and cancer. However, mice that were treated with SR9009/SR9011^25^, or another potent derivative SR10067^26^, all exhibited reduced wheel-running activities, implying that the treatment could lead to inactivity. Nobiletin is another small molecule that was shown to protect against obesity in HFD-fed *Clock*^*D19/D19*^ mutant mice, and amend glucose intolerance in diabetic *db/db* mice in a RORα/γ-dependent manner^27^. Nobiletin extended the median lifespan of mice under regular diet feeding and improved mitochondrial function via activating OXPHOS supercomplex-encoding genes^28^, which suggested that Nobiletin is an adequate option for supporting circadian clock robustness and healthspan. Small molecules that affect CRY1/2 stability were also found to be applicable in tuning circadian rhythm and alleviating metabolic diseases. CRY1/2 stabilizer KL001 and the derivatives were shown to lengthen the period and decrease circadian amplitude in vitro^29^, and proved to lower glucose intolerance in obese mice^31^. These results implied an intriguing point that both increasing and decreasing the circadian strength could be practical in treating glucose intolerance and obesity in genetic, or diet-provoked disease models. Nevertheless, reports of direct inspections of these circadian modulators in wild-type, aged mice for their rejuvenating activities remain limited. Also, it is important to note that the majority of these circadian-modulating compounds showed a clear phase-altering effect^32,33^, so their applications would consider tentative due to long-term phase mis-alignment is known to be deleterious to health^34–36^. For these reasons, we searched for small molecules that are able to promote circadian amplitude persistently without causing period change. From our chemical screen, we identified a small molecule ISX-9 that appeared to fulfill the pursuit. ISX-9 was originally found to facilitate neural stem cell conversion into neuron^37^. Here we found that ISX-9 can enhance PER2::LUC amplitude and BMAL1 activity via triggering Ca^2+^ influx and CaMKIIδ-mediated phosphorylation on BMAL1. Together with other effects found in ISX-9-treated mice, including higher PER2 expression in the suprachiasmatic nucleus (SCN), and improved sleep homeostasis, our results pointed out that ISX-9 could help to mitigate age-associated circadian rhythm disorder and metabolic dysregulation.

## Results

### Screen of circadian amplitude enhancers

**Fig. 1:**
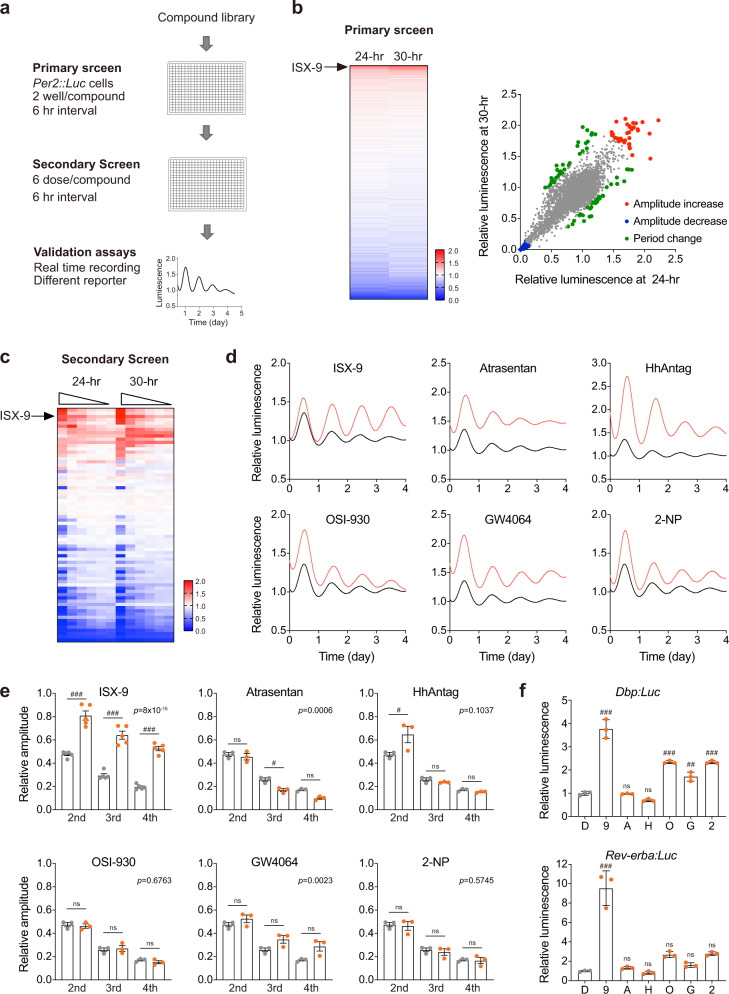
Small molecule screen identified ISX-9 as a circadian amplitude enhancer. **a** A schematic diagram of the compound screen procedures and validation. **b** Heatmap of the primary screen result of 6777 small molecules (left). Dots represent PER2::LUC bioluminescence at 24-h and 30-h time points, normalized to vehicle control. **c** Dose-dependent validation of 72 evident circadian-modulating compounds from the primary screen. Each compound was tested with threefold dilution series for six doses, with 10 μM as the highest concentration. **d** Real-time bioluminescence recording of synchronized *mPer2*^*Luc*^ MEF cells treated with indicated small molecules (red curve) that revealed amplitude enhancement activities from the screen. DMSO-treated oscillations were shown as control (black curve). **e** Amplitude evaluation of 4-day bioluminescence peaks responded to small molecule treatments. Amplitudes of each compound were normalized to its respective day-1 peak. All data were represented as mean ± SD. Two-way ANOVA with Bonferroni correction for multiple comparison was performed, with *p* value labeled on top. Significances between non-treated and treated cells in each day were shown (ns not significant, #*p* < 0.05, ###*p* < 0.001). **f** Changes of *Dbp:Luc* and *Rev-erbα:Luc* expressions responded to small molecule treatments relative to DMSO. One-way ANOVA with Bonferroni correction for multiple comparison was performed (ns not significant, ##*p* < 0.01, ###*p* < 0.001), and data were shown as mean ± SD.

To explore circadian modulators that exert amplitude-enhancing activity with better persistence and minimal disturbance to the period, we carried out a compound screen that included 8060 small molecules covering kinases, epigenetic factors, and GPCRs as targets in *mPer2*^*Luc*^ fibroblasts^38^ (Fig. 1a). To validate amplitude versus phase change in the highthroughput primary screen, we measured the luminescence level at 24-h and 30-h, respectively, in *mPer2*^*Luc*^ fibroblasts post drug treatment (Fig. 1b and Supplementary Fig. 1a). The two reads allowed to indicate amplitude modulators if finds consistent increasing or decreasing effects on luminescence level in both time points, or phase modulators if the two values are reversing with related to the untreated control, as represented by BS-181 for the latter case (Supplementary Fig. 1b, c). After eliminating those with a clear cell toxicity effects, we focused on the remaining 6777 compounds (Fig. 1b and Supplementary Data 1) and identified 270 amplitude enhancers that were able to increase PER2::LUC amplitude by 1.2-fold (Supplementary Data 1). Among them, ISX-9, Atrasentan, HhAntag, OSI-930, GW4064, and 2-NP were clear examples of raising PER2::LUC luminescence in the secondary screen (Fig. 1c). Real-time recording of PER2::LUC luminescence confirmed that the six candidates were able to increase PER2::LUC amplitudes without affecting the circadian period (Fig. 1d and Supplementary Fig. 1d). Notably, most candidates enhanced PER2::LUC amplitude evidently only in day 1, then showed substantial efficacy decline in the remaining 3 days. ISX-9 acted as the sole endurable amplitude enhancer over 4 days of testing (Fig. 1e). The amplitude-enhancing effect of ISX-9 was consistent, and applicable to other circadian gene promoter-driven luciferase reporters, including *Dbp:Luc* and *Rev-Erbα*:*Luc*, in both cases revealed with the best efficacy among the six candidates (Fig. 1f).

### ISX-9 augments circadian amplitude

**Fig. 2:**
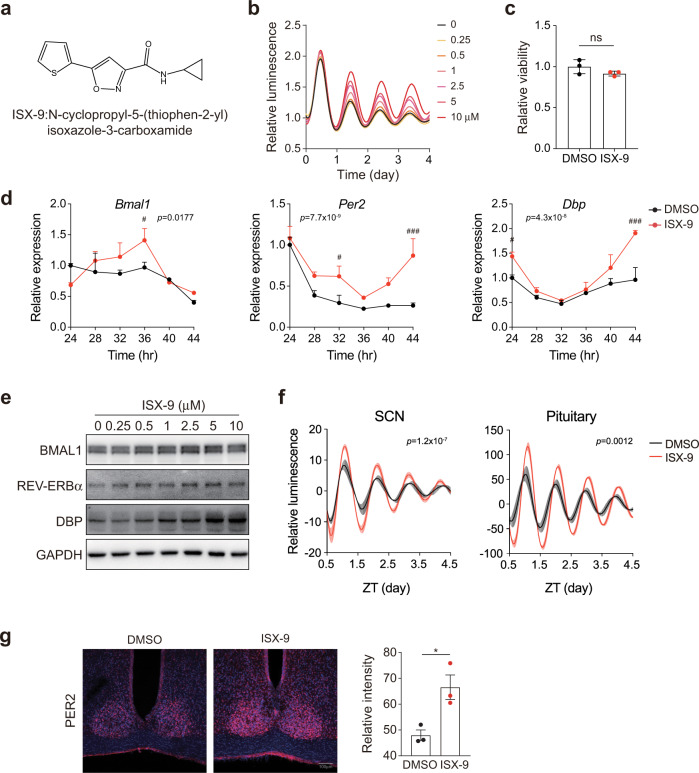
ISX-9 enhanced circadian amplitudes in culture cells and tissues. **a** Chemical structure of ISX-9. **b** Dose-dependent effects of ISX-9 in the *mPer2*^*Luc*^ MEFs. **c** Viability test of ISX-9 in MEF cells for 72 h. Unpaired Student’s *t*-test was used (ns not significant). **d** Quantitative PCR analyses of clock gene expressions in MEFs treated with vehicle or ISX-9 at different zeitgeber time points. Two-way ANOVA with Bonferroni correction for multiple comparison was performed, with *p* value labeled on top. Significances between DMSO and ISX-9-treated cells in each time point were shown (#*p* < 0.05 and ###*p* < 0.001), and data were shown as mean ± SD. **e** Immunoblots for BMAL1, REV-ERBα, and DBP levels upon increasing doses of ISX-9 treatment. **f** ISX-9 (10 μM) enhances PER2::LUC rhythms in SCN explants and pituitary from *mPer2*^*Luc*^ mice. Two-way ANOVA with Bonferroni correction for multiple comparison was performed with *p* value indicated on top, and data are shown as mean ± SEM. **g** Immunohistochemistry of PER2 level in the SCN from 6-month-old mice treated with DMSO or ISX-9 (20 mg/Kg) for 7 days. Quantification of the PER2 levels were shown (right), as mean ± SEM. Unpaired Student’s *t*-test was used (*p < 0.05).

ISX-9 is an isoxazole compound that was initially identified with neurogenic activity^37^ (Fig. 2a). In our test, we found ISX-9 elicited PER2::LUC amplitude in a dose-dependent manner (Fig. 2b), and showed low cell toxicity at 10 μM, the highest concentration in our examination (Fig. 2c). Agreed with the luciferase assays, ISX-9 augmented the levels of several clock transcripts including *Bmal1*, *Per2*, and *Dbp* (Fig. 2d and Supplementary Fig. 2a). DBP protein level was highly responsive to the ISX-9 dosage in comparison to BMAL1 and REV-ERBα (Fig. 2e), thus appeared to be a suitable marker to report ISX-9 effect in vitro. Interestingly, we found ISX-9 activity on DBP and PER2 inductions lessened in *Bmal1*^*+/−*^, and blunted in *Bmal1*^*−/−*^ MEFs, indicating that the amplitude-enhancing activity of ISX-9 is BMAL1-dependent (Supplementary Fig. 2b, c). ISX-9 also elicited higher circadian PER2::LUC oscillations in explants, including the central circadian oscillator suprachiasmatic nucleus (SCN) and pituitary (Fig. 2f), and mice that were administrated with ISX-9 revealed higher PER2 level in the SCN (Fig. 2g), collectively telling a dependable amplitude-enhancing effect of ISX-9 in vitro, ex vivo, and in vivo.

### ISX-9 ameliorates age-associated circadian disorders

**Fig. 3:**
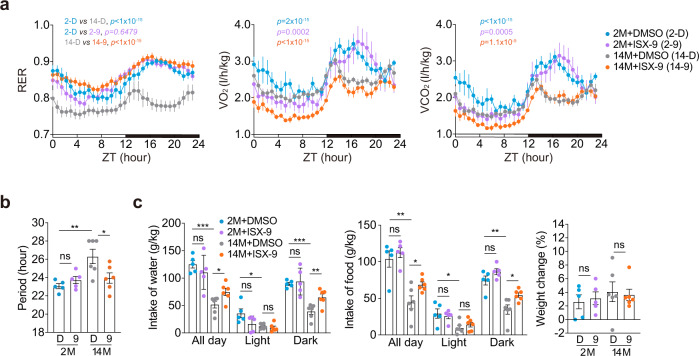
ISX-9 rejuvenated metabolic oscillations in middle-aged mice. **a** Respiratory exchange ratio (RER), oxygen consumption (VO_2_), CO_2_ production (VCO_2_) of male 2-month-old mice treated with DMSO (blue curve, *n* = 5) or ISX-9 (purple curve, *n* = 5) versus male 14-month-old mice with DMSO (gray curve, *n* = 6) or ISX-9 (orange curve, *n* = 6). All data were represented as mean ± SEM. Two-way ANOVA with Bonferroni correction for multiple comparison was performed, and the *p* values between indicated groups were listed on top. **b** Period of RER fluctuation from the metabolic cage data, processed with the software ClockLab. **c** Analysis of daily water intake, food consumption, and body weight change in the same mouse cohorts as in Fig. 3a. Unpaired Student’s *t*-test was used (ns not significant, *p < 0.05, **p < 0.01, ***p < 0.001), and data are shown as mean ± SEM.

To explore the metabolic-adjusting effect of ISX-9 in vivo, we further examined the metabolic rate in juvenile (2-month-old) and middle-aged (14-month-old) mouse cohorts. The juvenile mice exhibited clear respiratory exchange ratio (RER) oscillations diurnally and showed lower RER representing a resting/fasting state during daytime (Fig. 3a)^39,40^. The middle-aged group showed a less distinctive diurnal RER phenotype in the metabolic cage, with only a brief elevated RER indicating a feeding-associated carbohydrate utilization in the early night (Fig. 3a). ISX-9 administration increased the amplitude of RER in the middle-aged group, but did not alter RER amplitude in the juvenile group (Fig. 3a). Moreover, we found ISX-9 corrected the RER period from 26 to 24 h (Fig. 3b), and slightly promoted water-assessment and food intake during the dark time, but not affect body weight in the middle-aged group (Fig. 3c), and the effect can be extended to another test in an older 18-month-old cohort (Supplementary Fig. 3a–e). As ISX-9 treatment did not affect the juvenile cohort (Fig. 3a), the results indicated that ISX-9 acted selectively in ameliorating metabolic dysregulations associated with aging. The ISX-9 treatment was conducted before the metabolic cage housing, confirming a sustainable activity of ISX-9 in promoting circadian amplitude in vivo.

**Fig. 4:**
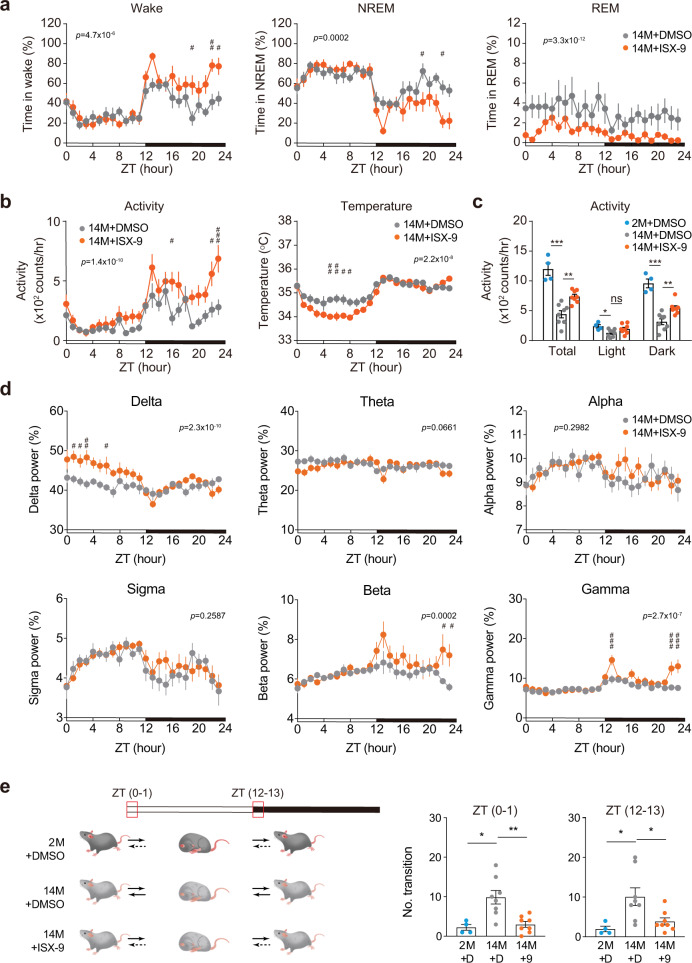
Diurnal electroencephalogram and physical activities prior to and post ISX-9 treatment in aged mice. **a** Percentage of time in wakefulness, NREM sleep, and REM sleep of male 14-month-old mice treated with DMSO (gray curve, *n* = 8) or ISX-9 (orange curve, *n* = 8). The bottom indicates the light-on period (white section, 07:00–19:00) and light-off period (black section, 19:00–07:00), respectively. **b** Diurnal activity and body temperature changes in the 24-h cycle of male 14-month-old mice treated with DMSO (gray, *n* = 8) or ISX-9 (orange, *n* = 8). **c** Comparison of diurnal activity changes in male 2-month-old mice treated with DMSO (blue, *n* = 4), 14-month-old mice treated with DMSO (gray, *n* = 8) or ISX-9 (orange, *n* = 8). **d** EEG power analysis in male 14-month-old mice treated with DMSO (gray curve, *n* = 8) or ISX-9 (orange curve, *n* = 8). The proportion of absolute powers during a circadian day for delta (0.5–4 Hz), theta (4–8 Hz), alpha (8–12 Hz), sigma (12–16 Hz), beta (16–32 Hz), and gamma (>32 Hz) were shown. **e** State transitions between wake and sleep stages in the first hour of light-on (ZT0–1) or light-off (ZT12–13). **a**, **b**, **d** All data were represented as mean ± SEM. Two-way ANOVA with Bonferroni correction for multiple comparison was performed, with *p* value labeled on top. Significances between DMSO and ISX-9-treated mouse cohorts in each time point were shown (#*p* < 0.05, ##*p* < 0.01, ###*p* < 0.001). **c**, **e** Unpaired Student’s *t*-test was used (ns not significant, *p < 0.05, **p < 0.01, ***p < 0.001), and data were shown as mean ± SEM.

To study whether ISX-9 could ameliorate sleep disorder, another phenotype that is associated with compromised circadian regulation^7,8^, we conducted a telemetric recording of electroencephalogram (EEG) to score sleep status in a young versus old manner. The differences in wake and NREM levels were found most evidently during night time by comparing 2-month-old and 14-month-old mice. The latter exhibited partial awake, which correlated well with higher NREM sleep in the night time (Supplementary Fig. 4a). Together with the data including largely reduced physical activity, shallowed body temperature fluctuation (Supplementary Fig. 4b), lowered beta/gamma, and blunted delta/theta oscillations (Supplementary Fig. 4c), the results pointed out a collectively depressed situation in circadian amplitudes during aging. To test whether ISX-9 could entrain the circadian-programmed sleep cycle, we dosed the same group of mice with 20 mg/Kg ISX-9 for 7 days, then stopped the treatment for another 7 days before the EEG recoding. The treatment sharply promoted wake in the early night and sustained a higher wake state in the remaining dark period, at the same time lowered both REM and NREM sleep in the active period (Fig. 4a). The treated middle-aged mice also displayed higher physical activities during night time, conversely exhibited lower body temperature during the day (Fig. 4b, c), indicating that ISX-9 has tuned the middle-aged mice to a more alert, active status during awake, and stayed better rested during sleep. We noted the treated middle-aged mice with higher delta power in the resting period, and higher gamma power in the awake period, both in advance, revealed improved circadian homeostasis in brain oscillations (Fig. 4d). Indeed, by counting the transitions in the early stages of the awake-sleep cycle at light-on (ZT0-1) and light-off (ZT12–13), we found ISX-9 markedly suppressed dysregulated switches in the middle-aged group, by which rendered more stable sleep and wake homeostatic states, as observed in healthy juvenile mice (Fig. 4e).

### CaMKIIδ mediates ISX-9 effect via BMAL1

**Fig. 5:**
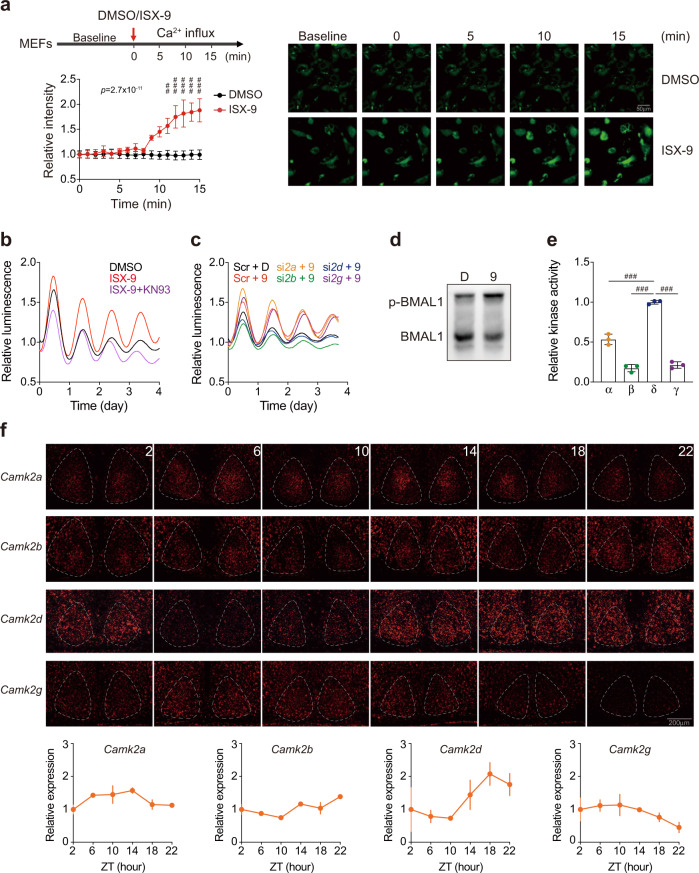
ISX-9 triggered Ca^2+^ influx and BMAL1 phosphorylation in a CaMKIIδ-dependent manner. **a** Assay scheme for the Ca^2+^ influx measurement (left), and Fluo-4 AM fluorescence change upon DMSO (black curve) or 10 μM ISX-9 (orange curve) treatment in MEF cells. Representative images were shown at indicated time points (right). Scale bar, 50 μm. Two-way ANOVA with Bonferroni correction for multiple comparison was performed, with *p* value labeled on top. Significances between DMSO and ISX-9-treated cells in each time point were shown (##*p* < 0.01, ### *p* < 0.001), and data were shown as mean ± SD. **b** PER2::LUC oscillations under DMSO (black curve), ISX-9 (red curve), and ISX-9 with CaMKII inhibitor KN93 (magenta curve) treated conditions. **c** PER2::LUC oscillations under DMSO, ISX-9, and individual knockdown of *Camk2a*, *Camk2b*, *Camk2d,* and *Camk2g* with ISX-9 treatment. **d** Phos-tag gel analysis for BMAL1 phosphorylation level in DMSO (D), or 10 μM ISX-9 (9) treated MEF cells. **e** Purified CaMKII α, β, γ, and δ were subjected to in vitro kinase assay using purified BMAL1 as the substrate. The highest activity was set as 1 to facilitate comparison among isoforms. One-way ANOVA with Bonferroni correction for multiple comparison was performed (###*p* < 0.001), and data were shown as mean ± SD. **f** Single-molecule RNA fluorescence in situ hybridization of *CamK2a, b, d*, and *g* in the SCN from male 2-month-old wild-type mice (*n* = 3) at indicated zeitgeber time. Expression levels in SCN were quantified and normalized to the first time point as 1, data are shown as mean ± SEM.

ISX-9 was identified to promote neuronal differentiation through Ca^2+^ signaling^37^. We first followed the intracellular Ca^2+^ level by Fluo-4 AM binding assay in MEFs, and confirmed that ISX-9 was able to trigger Ca^2+^ influx rapidly in our tests (Fig. 5a). Consistent with the previous report, we also found that the ISX-9 effect was mediated by CaMKII activity, as the CaMKII-specific antagonist KN93 fully abolished the PER2::LUC enhancing effect of ISX-9 (Fig. 5b). To test whether CaMKII agonist could augment the amplitude of circadian oscillations without period change, we applied another small molecule, Isoproterenol, that was reported to stimulate β1-adrenergic receptor thus trigger Ca^2+^ influx and CaMKII activity^41^. We noted that Isoproterenol can also increase PER2::LUC amplitude, though with subtle efficacy when compared to ISX-9 (Supplementary Fig. 5a). Similar to ISX-9, Isoproterenol did not show period altering effect (Supplementary Fig. 5b). Among the four CaMKII isoforms^42^, we noted that *Camk2b* and *Camk2d* knockdowns retracted the ISX-9 effect evidently in real-time PER2::LUC recordings, but not in *Camk2a* and *Camk2g* knockdowns (Fig. 5c). Given that ISX-9 showed BMAL1 dependency in prompting DBP and PER2::LUC levels (Supplementary Fig. 2b, c), and triggered BMAL1 phosphorylation (Fig. 5d), we next examined the potential CaMKII activities on BMAL1 phosphorylation biochemically, and compared the differences among CaMKII isoforms. CaMKIIδ exhibited the highest activity on purified BMAL1 in the kinase assay (Fig. 5e). Together with the *Camk2d* knockdown result on PER2::LUC expression, the data implicated that CaMKIIδ was critical to mediate the ISX-9 effect through BMAL1. These collectively proposed an intriguing loop of connecting ISX-9, Ca^2+^ signaling, and BMAL1 phosphorylation for modulating the circadian amplitude. Interestingly, single-molecule RNA fluorescence in situ hybridization (smFISH) indicated that *Camk2d* distribution in the mouse brain was uniquely more abundant in subcortical regions and the hypothalamus (Supplementary Fig. 6), and showed a clear rhythmic expression that peaked at ZT18 in the SCN (Fig. 5f), jointly fit well with the anticipated role in circadian regulation. Other CaMKII isoform transcripts, such as *Camk2a* and *Camk2b* showed subtle oscillations and lower expressions in the SCN, and distributed more abundantly in the striatum and cortex (Supplementary Fig. 6). Furthermore, *Camk2d* was considerably reduced in the SCN of aged mice (Supplementary Fig. 7a), and ISX-9 improved *Camk2d* expression (Supplementary Fig. 7b), the results put forward the situation that ISX-9 helped to re-boost the CaMKIIδ level, accordingly the CaMKIIδ-mediated phosphorylation of BMAL1, in turn, reverted the circadian amplitude decline resulted from aging. Expression levels of *Camk2a* and *Camk2b* did not alter in old SCN, nor respond to the ISX-9 treatment (Supplementary Fig. 7). Of note, it seemed *Camk2g* expression was anti-phasic (peaked at ZT6) to *Camk2d* (Fig. 5f), and *Camk2g* showed slight up-regulation in the old SCN (Supplementary Fig. 7a). Whether the later indicated a marker of aging SCN or a compensatory effect to respond the decay of *Camk2d* would be intriguing questions to investigate afterward.

### CaMKIIδ regulates circadian amplitude via phosphorylating BMAL1

**Fig. 6:**
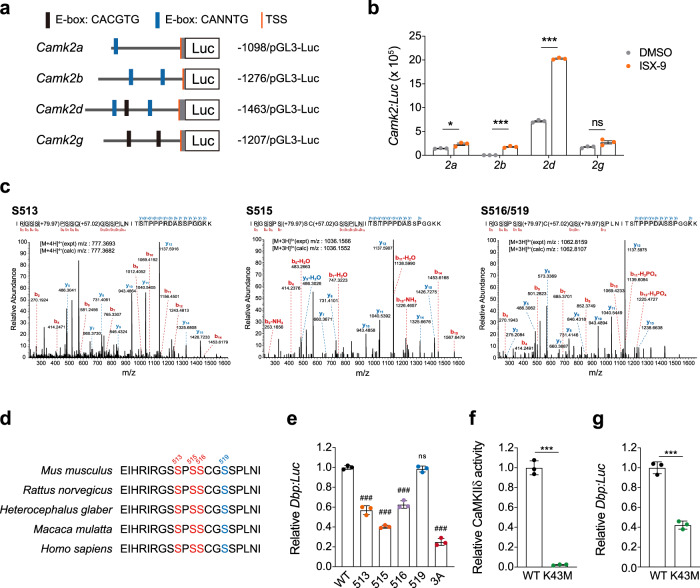
ISX-9 prompted BMAL1 activity through S513/515/516 phosphorylation. **a** Schematic diagram of E-box containing *mCamk2* promoter constructs. **b** *Camk2*:Luc bioluminescence assay in N2a cells treated with DMSO or 10 μM ISX-9. **c** Mass spectrometry identification of potential BMAL1 phosphorylation sites responded to ISX-9 treatment in N2a cells. **d** Amino acid sequence alignment of BMAL1 among the indicated species, S513, S515, and S516 were marked in red, and S519 in blue. **e** *Dbp*:Luc bioluminescence assay with expressing BMAL1 WT or mutants in N2a cells. One-way ANOVA with Bonferroni correction for multiple comparison was performed (ns, not significant, ### *p* < 0.001), and data were shown as mean ± SD. **f** In vitro kinase assay of purified CaMKIIδ WT and K43M mutant, using BMAL1 as the substrate. **g** *Dbp*:Luc bioluminescence assay with expressing CaMKIIδ WT or K43M mutant. **b**, **f**, **g** Unpaired Student’s *t*-test was used (ns not significant, *p < 0.05, ***p < 0.001), and data were shown as mean ± SD.

**Fig. 7:**
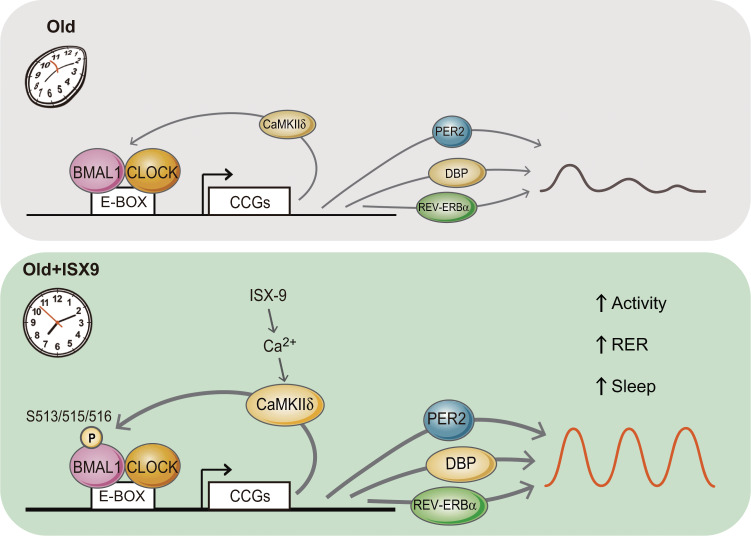
Model of ISX-9 effect through CaMKIIδ-mediated activation of BMAL1 for circadian amplitude enhancement. Aging is commonly associated with the declined circadian amplitude of clock gene expressions, decreased locomotor activity, and reduced sleep homeostasis. ISX-9 facilitates Ca^2+^ influx further triggers BMAL1 S513/515/516 phosphorylation in a CaMKIIδ-dependent manner in aging cells. The effect, in turn, upregulates the expression clock outputs, including PER2, DBP, and REV-ERBα, and promotes locomotor activity, daily RER fluctuation, and sleep homeostasis in middle-aged mice. Given that CaMKIIδ is a clock-controlled protein that also dampens with aging, ISX-9 stimulation helps to intensify a positive feedback loop through CaMKIIδ to modulate BMAL1 activity, therefore, reverts the circadian amplitude decay caused by aging.

Given that *Camk2d* expression was rhythmic and could be triggered by ISX-9, we searched for putative E-box sequences in the *Camk2* promotors. We found all isoforms contain E-box elements, including the canonical E-box sequence (CACGTG) identified in *Camk2d* and *Camk2g* promotors (Fig. 6a). Using promotor reporting assay in neuroblastoma N2a cells with comparing CLOCK:BMAL1 co-expression to non-supplemented basal condition, we found *Camk2d* promotor was most responsive to CLOCK:BMAL1 activation with a 6.8-fold increase in *Camk2d:Luc* expression (Supplementary Fig. 8a), and the induction was not due to dexamethasone synchronization in the test (Supplementary Fig. 8b). ISX-9 could further boost the expression of *Camk2d:Luc* to the highest level with CLOCK:BMAL1 co-expression (Fig. 6b), the results indicated a preferable expressional response of *Camk2d* to ISX-9 that was consistent with the observation in aged SCN (Supplementary Fig. 7b). Furthermore, we found *Camk2d:Luc* expressed rhythmically in MEFs (Supplementary Fig. 8c), collectively the data verified that *Camk2d* is an authentic clock-controlled gene. Mass spectrometry identified BMAL1 serine 513, 515, 516, and 519 (Fig. 6c), four highly conserved serine residues among different species (Fig. 6d), to be major phosphorylated sites that responded to ISX-9 treatment. Mutation of either S513, S515, or S516 residue to alanine led to 40–60% decrease in BMAL1 activity of driving the expression of BMAL1-dependent *Dbp:Luc*, but not S519A in the same test (Fig. 6e). The S513A/S515A/S516A triple mutation produced an even substantial impact on BMAL1 activity, suggesting that the three serine residues are important for BMAL1 activation. Consistent with the idea, the CaMKIIδ catalytic mutation K43M (an equivalent design to mimic CaMKIIα-K42M) (Supplementary Fig. 9a)^42,43^ with confirmed blunt kinase activity on a substrate peptide (Supplementary Fig. 9b)^44^ and purified BMAL1 (Fig. 6f), led to similar downgrade effect in *Dbp:Luc* expression in N2a cells (Fig. 6g). Under CLOCK/BMAL1/CaMKIIδ co-expression conditions, BMAL1-S513A/S515A/S516A and CaMKIIδ-K43M showed a similar defect in driving *Dbp:Luc* expression in comparison to their wild-type counterparts (Supplementary Fig. 9c), these results highlighted again a CaMKIIδ-associated regulation of circadian amplitude via BMAL1. All told, our data suggested that CaMKIIδ, as a clock output, comprised a positive feedback loop in modulating BMAL1 activity, and their decay caused by aging could be rejuvenated by CaMKII agonist ISX-9 (Fig. 7).

## Discussion

ISX-9 was shown to exert interesting activities, initially identified as a neurogenic chemical that could convert neural stem cells into neurons^37^. In a later work, ISX-9 was further revealed to be able to reprogram fibroblasts into functional neurons along with a BET family bromodomain inhibitor, I-BET151^45^. ISX-9 was then proposed in a few applications related to neurogenesis, for instance, to potentiate cell proliferation and neuronal commitment in the dentate gyrus in vivo^46^. Interestingly, besides the primary role as a neurogenesis inducer, ISX-9 was reported to be useful in treating neuropsychiatric conditions, such as lowering the relapse of methamphetamine abuse^47^ and fear response in ethanol-dependent rat model^48^. In an age-related perspective, ISX-9 was shown to rescue the loss of cortico-striatal synapse, thus improving cognition in Huntington’s disease model R6/2 strain^49^. ISX-9 application can be extended to other peripheral tissues in addition to the brain, for example, to promote pancreatic β-cell survival against apoptosis^50^.

Here we proposed ISX-9 as a small molecule-based intervention to persistently magnify the circadian amplitude, which is commonly tapered in an aging-sensitive fashion^19,21^. The amplitude decline is partly due to, as we proposed here, the decay of CaMKIIδ, thus restricting its capacity to lift BMAL1 activity through S513/S515/S516 phosphorylation. BMAL1 phosphorylation has been demonstrated to play both promotive^51,52^ and suppressive^53,54^ roles, judged by the serine/threonine sites and the subsequent outcomes to regulate BMAL1 transcriptional activity of BMAL1 stability. Here we add S513/S515/S516 as a novel regulatory cluster on BMAL1 to respond to the CaMKIIδ-mediated activation. The mode is likely suitable for setting circadian rhythms in the central circadian oscillator SCN, as CaMKIIδ expresses more abundantly in the SCN, and SCN shows clear calcium level change diurnally^55^. Future works regarding the understanding of BMAL1 phosphorylation, with details on the corresponding residues combining temporal information, would be important to support versatile designs for modulating circadian amplitude and phase.

Our finding is coherent with other evidence which demonstrated the importance of calcium signaling and CaMKII activity in regulating circadian rhythms. Calcium influx was recognized as an important signal for circadian rhythm generation in the SCN^56–60^, and mice carrying CaMKIIα-K42R mutation was shown to exhibit a prolonged period phenotype^61^. Our study took a step forward to show that sensitizing Ca^2+^ influx by ISX-9 is a convenient way to replenish another CaMKII participant, CaMKIIδ oscillation, thus adjusting the circadian amplitude in aging cells. Together with other positive activities discussed earlier, the use of ISX-9 seemed to be promising in defying age-related diseases, markedly also in neurological disorders. Whether these beneficial effects were all associated with improved circadian functions, and whether the advantageous outcomes observed in the current study could be extended to female or older subjects (as we only investigated the ISX-9 effect in 14–18-month-old male mice), are interesting topics to examine in the future.

## Methods

### Reagent

Small molecules that were elected for further oscillation tests were purchased from the following companies: ISX-9 from Selleck; GW4064, OSI-930, Atrasentan, Forskolin, and KN93 from Med Chem Express; 2-NP and HhAntag from TargetMol. Antibodies were purchased from the following resources: anti-BMAL1 (14020, Cell Signaling Technology), anti-DBP (12662-1-AP, ProteinTech), anti-PER2 (13168, ABclonal), anti-REV-ERBα (13418, Cell Signaling Technology), anti-GAPDH (60004, ProteinTech), HRP-conjugated goat anti-rabbit secondary antibody (1706515, Bio-Rad), and HRP-conjugated goat anti-mouse secondary antibody (1706516, Bio-Rad).

### Cell line

HEK293T, NIH-3T3, and N2a cell lines were obtained from ATCC. MEF cells were isolated from E13.5d embryos of wild-type, *Bmal1*^*−/−*^ or *mPer2*^*Luc/+*^ mice. Cells were cultured in DMEM (Invitrogen) with 10% FBS and 100 U/ml penicillin/streptomycin, and maintained at 37 °C with 5% CO_2_ in a humidified environment. All cell lines were examined with a PCR-based method (MP0035, Sigma) to ensure mycoplasma-free.

### Animal strain

Reporter mouse strain *mPer2*^*Luc*^ (006852)^38^ and *Bmal1*-floxed conditional strain (007668)^62^ were obtained from the Jackson Laboratory and maintained in a C57BL/6 J background. Mice were group housed in a specific pathogen-free facility under a 12:12 h light-dark cycle at 22 ± 2 °C, and animal experiments were conducted in male mice in accordance with protocols approved by the institutional Biomedical Research Ethics Committee, Shanghai Institute of Nutrition and Health and Center for Excellence in Brain Science and Intelligence Technology, Chinese Academy of Sciences.

### Plasmid

For luciferase-based expression assays, mouse *Camk2a/b/d/g*, *Dbp*, and *Rev-erbα* promoter fragments were PCR amplified from mouse genomic DNA and then cloned into pGL3-Basic-luc vector (E1751, Promega) via *Mlu*I and *Bgl*II sites (primer information see Supplementary Table 1). Constructs for overexpressing *Bmal1*, FLAG-*Bmal1*, FLAG-*Camk2a, b, d*, and *g* were cloned via *Not*I and *Mlu*I sites into a pCAGGS vector with FLAG-tag fusion. *Bmal1* mutants (S513A, S515A, S516A, S519A, and S513A/S515A/S516A) and *Camk2d* K43M mutant were generated using PCR-based site-directed mutagenesis (primer information list in Supplementary Table 1), then subcloned into the same pCAGGS backbone.

### Small molecule screen

Primary small molecule screen (Natural Products Library, NIH Clinical Collection LOPAC Collection and The Spectrum Collection from Sigma-Aldrich; New compound library, Epigenetics library and GPCR & G Protein library from Selleck; Approved drug screening library and Inhibitor library from TargetMol; Stem cell regulator library from Merck; Neurotransmitters library from Tocris Bioscience; Exclusive new compound library from MCE; and Nuclear Receptor Ligand agonists or antagonists Library from Enzo Life Sciences) was carried out in 384-well plates, with ~3000 *mPer2*^*Luc*^ MEF cells seeded 48 h prior to the chemical applications at 10 μM dosage. Luciferase activities were measured at two time points, 24-h and 30-h post chemical treatment, respectively, using the ONE-Glo™ luciferase assay system (E6120, Promega). After chemiluminescence measurement, cell viability was determined by alamarBlue assay (Invitrogen). The summary of the relative compound effect was listed in Supplementary Data 1. The screen was performed in the Chemical Biology Core Facility, Center for Excellence in Molecular Cell Science, CAS.

### Real-time bioluminescence recording

For the real-time recording of PER2::LUC bioluminescence, ~2.5 × 10^5^ *mPer2*^*Luc*^ MEF cells were seeded and then cultured for 48 h with DMEM in 3.5 cm dishes until confluent. The cells were then synchronized with 200 nM dexamethasone for 1 h, before the bioluminescence recording in serum-free DMEM medium supplemented with 200 μM luciferin (A5030, Tokyo Chemical Industry) together with indicated small molecules at 10 μM or indicated concentration. For *Camk2d:Luc* oscillation recording, pGL3 reporter plasmid containing mouse *Camk2d* promoter was transfected (1 μg) via Lipofectamine 3000 (Invitrogen) into 2.5 × 10^5^ MEF cells, then cultured for 48 h before 200 nM dexamethasone synchronization then bioluminescence recording. The cultures were recorded in a light-tight chamber containing photon multiplier tube recording (PMT) detector assemblies (LumiCycle 32, Actimetrics) for continuous 4 days. Data including amplitude and period results, were processed with the LumiCycle Analysis software (Actimetrics).

### Tissue culture for bioluminescence recording

SCN slices and recordings were conducted as described in ref. ^63^. In brief, brains were harvested from 6-month-old *mPer2*^*Luc*^ mice, then the SCN slices were sectioned via McIlwain Tissue Chopper (TC752, Cavey Laboratory Engineering Co. Ltd) into 300-μm thickness. SCN slices were cultured on a membrane (PICM0RG50, Millipore) in a 1.5 ml recording medium (1x DMEM, 1x B27 supplement, 4.2 mM NaHCO_3_, 10 mM HEPES, 1X penicillin/streptomycin and 200 μM luciferin) together with DMSO or ISX-9 (10 μM), then detected by continuous PMT recording (LumiCycle 32, Actimetrics). Pituitary was cultured and recorded under the same conditions as for SCN.

### Quantitative RT-PCR analysis

Total RNA was extracted using RNeasy Mini Kit (Qiagen) according to the manufacturer’s instructions. Approximately 3 μg RNA was applied for cDNA synthesis using Omniscript RT Kit (205111, Qiagen). Real-time PCR reactions were assembled with the use of the SYBR Green PCR Kit (208054, Qiagen), then analyzed in an ABI ViiATM 7. The QuantStudioTM Real-Time PCR Software was used to determine gene expression levels and normalized to a ribosomal reference gene, *Rpl19*. Primer sequences are listed in Supplementary Table 2.

### Immunoblot

Cell cultures were lysed in chilled lysis buffer (50 mM Tris at pH 7.5, 150 mM NaCl, 5 mM MgCl_2_, 0.5% Nonidet P-40 and 1% Triton X-100) supplemented with protease inhibitors (118735800, Sigma-Aldrich) and phosphatase inhibitors (4906837001, Roche). The lysates were measured for protein concentration by Bradford assay, then adjusted for equal protein content before immunoblot analysis with antibodies described above. To prepare the Phos-tag gel electrophoresis, cell lysates were resolved in 4% acrylamide gels containing 40 μM acrylamide Phos-tag ligand (F4002, APExBIO) and 80 μM MnCl_2_.

### Immunohistochemistry

Male mice at indicated ages were euthanized, then perfused with 4% paraformaldehyde, subsequently cryoprotected in 30% sucrose before being embedded in OCT (4583, SAKURA). Coronal brain sections were collected by using a cryostat (CM1850, Leica) at 30-μm thickness, then treated with 3% H_2_O_2_ to quench residual peroxidase activity in samples. Followed by antigen retrieval step via 0.05% trypsin treatment for 5 min at room temperature, brain sections were blocked with TNB blocking buffer (100 mM Tris-HCl, pH 7.5, 150 mM NaCl, 10% FBS) prior to the incubation of primary anti-PER2 antibodies with 1:300 dilutions at 4 °C for overnight. After being washed with TNT buffer (100 mM Tris-HCl, pH 7.5, 150 mM NaCl, 0.05% Tween 20) three times, HRP-conjugated anti-rabbit secondary antibodies (1:1000) were applied to the sections and proceeded incubation for 1.5 h at room temperature. After TNT buffer washes three times, brain sections were incubated with fluorophore-labeled tyramide substrate (TSA plus Cyanine3 kit, PerkinElmer) and DAPI (10 ng/ml, Sigma-Aldrich), then visualized by confocal microscopy (Cell Observer, ZEISS). The microscopy results were analyzed and quantified by ImageJ.

### Indirect calorimetry

Indirect calorimetry in a metabolic cage was performed with the CLAMS-16 system (Columbus Instruments). 2-, 14-, and 18-month-old male mouse cohorts were treated with ISX-9 (20 mg/Kg) or DMSO for 7 days before housing in the metabolic cages. Mice were singly housed and allowed for accommodation for 2 days in the metabolic cage before measurements. Standard chow and water were provided ad libitum throughout the experiment. Measurements of VO_2_, VCO_2_, food intake, water access, and activity were recorded every 10 min for up to 3 consecutive days in a 25 °C cubicle, under a 12:12 h light-dark cycle. Physical activities and respiratory exchange ratio (RER = VCO_2_/VO_2_) were calculated via software CLAX (Columbus Instruments).

### Electroencephalography recording and EEG data analysis

Mouse EEG recording was carried out by HD-X02 telemetry implants (DSI PhysioTel™). Mice were deeply anesthetized by single intramuscular dosing of 5 mg/kg pentobarbital before implantation surgery. The two EEG electrodes were screwed into the skull 1 mm lateral, 1 and 3 mm posterior to the Bregma, respectively. After surgery, mice were intraperitoneally injected with 5% glucose solution (40 μl/gram body weight) for 7 days to facilitate recovery. Activity, body temperature, EEG, and EMG signals were collected by the RPC-1 receiver connected to an MX2 hub and a computer. The recordings were carried out under a normal light-dark cycle for 2 days as a pretreated baseline. Male mice at indicated age (2- and 14-month-old) were injected intraperitoneally with DMSO for 7 days, then waited for 7 days before the 3-day recording of EEG and EMG as the control data. Finally, the same cohorts of mice were injected with ISX-9 (20 mg/Kg) for 7 days, stayed untreated for 7 days, then proceeded to 3-day EEG and EMG recording. NeuroScore 3.2.0 (DSI) was applied for mouse EEG sleep analysis to summarize awake, REM, and NREM sleep stages, and identify the day-night sleep-wake transitions scored in 10-s epochs. EEG powers (δ: 0.5–4 Hz, θ: 4–8 Hz, α: 8–12 Hz, σ:12–16 Hz, β: 16–32 Hz, γ: >32 Hz) with 1-h average data points were shown^64,65^.

### In vitro Ca^2+^ imaging

MEF cells (2 × 10^5^) were seeded on glass bottom culture dishes (801002, NEST) 24 h prior to the incubation of Fluo-4 AM (2 μM, Molecular Probes) in serum-free DMEM for 45 min at 37 °C. After the cleaning of unbound dye by PBS rinse twice, the cells were measured for baseline emission (excitation 488 nm, emission 520 nm) for 15 min. The Ca^2+^-influx recording was started at DMSO or 10 μM ISX-9 addition (time 0). Images were collected at 1 min intervals for 15 min by fluorescence microscopy (Cell Observer, ZEISS).

### siRNA knockdown

Silencer oligonucleotides for mouse *Camk2a/b/d/g* and scramble controls were purchased from GenePharma (Scramble: UUCUCCGAACGUGUCACGU; si*Camk2a*: CAACAUCGUCCGACUACAU; si*Camk2b*:GAAGAGCUGCGCCCUGGUU; si*Camk2d*: GAAGCCAAAGACCUCAUCA; si*Camk2g*: GUAGAGUGCUUACGCAAAU). MEF cells were transiently transfected with siRNA oligonucleotides using Lipofectamine 3000 (Invitrogen) and then subjected to real-time bioluminescence recording as described above.

### Protein purification and kinase assay

For BMAL1 and CaMKII purifications, pCAGGS plasmids (5 μg) encoding FLAG-*Bmal1*, FLAG-*Camk2* isoforms, and mutants were transfected individually into 2 × 10^6^ HEK293T cells. After expressing for 48 h, cells were harvested and lysed in 400 μl lysis buffer containing protease inhibitor (Roche). Approximately 3 mg of cell lysates were incubated with 20 μl anti-FLAG M2 magnetic beads (Sigma) for 4 h at 4 °C, then cleaned the impurities via TBS buffer (50 mM Tris at pH 7.5, 150 mM NaCl) washes three times, before the elution by 100 μl of 3X FLAG peptide (150 ng/μl, Sigma). Kinase assays were carried out with a Universal kinase activity kit (EA004, R&D Systems) at 1:100 CaMKII to Autocamtide-2 Peptide (sc-3029, Santa Cruz Biotechnology), or 1:4 CaMKII to BMAL1 ratio, according to the manufacturer’s instruction, finally measured the absorbance of reactions at 620 nm.

### Single-molecule RNA in situ hybridization

Single-molecule in situ hybridization was performed with the RNAscope Multiplex Fluorescent v2-kit (Advanced Cell Diagnostics) according to the manufacturer’s instructions. Briefly, mice were euthanized, perfused with 4% paraformaldehyde, then the brains were carefully removed and post-fixed in 4% paraformaldehyde for overnight before the cryoprotection procedure in 30% sucrose-1X PBS at 4 °C. After embedding with OCT medium, 30-μm coronal brain sections were collected, followed by dehydration, peroxidase activity quenching, and target retrieval steps via Protease Plus solution (322381, Advanced Cell Diagnostics). After washes with H_2_O, sections were incubated with either *Camk2a, 2b, 2d, 2g* target probe (445231, 453601, 508941, and 522071, respectively, Advanced Cell Diagnostics, 1:300 dilution) at 40 °C for 2 h in HybEZ oven. Amplification, horseradish peroxidase incorporation, and tyramide stain steps were conducted as described in the user manual (323100-USM, Advanced Cell Diagnostics), followed by DAPI counterstain and fluorescence microscopy (VS120, OLYMPUS). The images were analyzed and quantified by ImageJ.

### Luciferase assay

N2a cells were seeded into 24-well dishes at 8 × 10^4^ cells per well density 24 h before transfections. *Bmal1/Clock* (200/200 ng), or *Camk2* (200 ng) overexpression with reporter plasmids (100 ng) were transfected per well with Lipofectamine 3000 (Invitrogen). After 24 h, cells were synchronized with 100 nM dexamethasone for 15 min, followed by the treatment with DMSO or 10 μM ISX-9 in DMEM for 24 h. Cells were harvested and determined for cell number, then ~1 × 10^5^ cells were lysed in 100 μl 1X Glo Lysis Buffer (E266A, Promega). About 5 μl lysate was mixed with 100 μl substrate (ONE-Glo™ luciferase assay system, E6120, Promega) for luciferase measurement (Lumat LB 9508, BERTHOLD).

### Immunoprecipitation and mass spectrometry

N2a cells were transfected with a pCAGGS-FLAG-*Bmal1* plasmid for the expression of FLAG-BMAL1 for 24 h with treating DMSO or 10 μM ISX-9. About 3 × 10^7^ cells were then harvested and lysed with 1200 μl chilled lysis buffer containing protease and phosphatase inhibitors (Roche). Approximately 10 mg of cell lysates were incubated with 60 μl anti-FLAG M2 magnetic beads (Sigma) for 4 h at 4 °C. The beads were then washed with TBS buffer (50 mM Tris at pH 7.5, 150 mM NaCl) three times, before the elution by 300 μl of 3X FLAG peptide (150 ng/μl, Sigma). The eluted FLAG-BMAL1 from ISX-9-treated or DMSO control were compared via immunoblot, before the analysis via mass spectrometry (LC-MS/MS). The identification of BMAL1 phosphorylation sites was carried out by first enriching the phospho-peptides with TiO_2_ beads, before the separation with an Easy-nLC 1200 nano HPLC (Thermo Fisher Scientific). Tandem mass spectrometry (MS/MS) analysis was performed with a Q Exactive Orbitrap mass spectrometer (Thermo Fisher Scientific) in National Facility for Protein Science in Shanghai.

### Statistics and reproducibility

An individual in vitro experiments were performed at least three times unless otherwise indicated. Animal experiments were independently repeated at least two times with consistent results. Numbers are indicated in the figure legends. Results are presented as mean ± SD or mean ± SEM, and statistical analyses were performed using Prism version 7.0 (GraphPad) with unpaired two-tailed Student’s *t*-test (statistical significances are labeled as ns not significant; *p < 0.05; **p < 0.01; ***p < 0.001), one-way ANOVA, or two-way ANOVA with Bonferroni correction (statistical significances are labeled as ns, not significant, #*p* < 0.05, ##*p* < 0.01, ###*p* < 0.001) as indicated in legends.

### Reporting summary

Further information on research design is available in the Nature Research Reporting Summary linked to this article.

## Supplementary information

Supplementary Information

Description of Additional Supplementary Files

Supplementary Data 1

Supplementary Data 2

Reporting Summary

## Data Availability

The small molecule screen results are available in Supplementary Data 1. The source data for graphs, charts, and immunoblots with the paper is available in Supplementary Data 2. The mass spectrometry proteomics data have been deposited to the ProteomeXchange Consortium (http://proteomecentral.proteomexchange.org) with the dataset ID PXD034339. The 18 plasmids associated with this study have been deposited to Addgene with the ID 186818-186835. All other data that support the finding of this study are available from the corresponding author upon reasonable request.

## References

[1] Asher G, Sassone-Corsi P (2015). Time for food: the intimate interplay between nutrition, metabolism, and the circadian clock. Cell.

[2] Bass J, Takahashi JS (2010). Circadian integration of metabolism and energetics. Science.

[3] Bass J, Lazar MA (2016). Circadian time signatures of fitness and disease. Science.

[4] Paschos GK, FitzGerald GA (2010). Circadian clocks and vascular function. Circ. Res..

[5] Marcheva B (2010). Disruption of the clock components CLOCK and BMAL1 leads to hypoinsulinaemia and diabetes. Nature.

[6] Fu L, Pelicano H, Liu J, Huang P, Lee C (2002). The circadian gene Period2 plays an important role in tumor suppression and DNA damage response in vivo. Cell.

[7] Mattis J, Sehgal A (2016). Circadian rhythms, sleep, and disorders of aging. Trends Endocrinol. Metab..

[8] Sehgal A, Mignot E (2011). Genetics of sleep and sleep disorders. Cell.

[9] Qiu PY (2019). BMAL1 knockout macaque monkeys display reduced sleep and psychiatric disorders. Natl Sci. Rev..

[10] Souetre E (1989). Circadian rhythms in depression and recovery: evidence for blunted amplitude as the main chronobiological abnormality. Psychiatry Res..

[11] Chung S (2014). Impact of circadian nuclear receptor REV-ERBalpha on midbrain dopamine production and mood regulation. Cell.

[12] Li JZ (2013). Circadian patterns of gene expression in the human brain and disruption in major depressive disorder. Proc. Natl Acad. Sci. USA.

[13] Landgraf D (2016). Genetic disruption of circadian rhythms in the suprachiasmatic nucleus causes helplessness, behavioral despair, and anxiety-like behavior in mice. Biol. Psychiatry.

[14] Musiek ES, Holtzman DM (2016). Mechanisms linking circadian clocks, sleep, and neurodegeneration. Science.

[15] Kondratova AA, Kondratov RV (2012). The circadian clock and pathology of the ageing brain. Nat. Rev. Neurosci..

[16] Chang HC, Guarente L (2013). SIRT1 mediates central circadian control in the SCN by a mechanism that decays with aging. Cell.

[17] Liu F, Chang HC (2017). Physiological links of circadian clock and biological clock of aging. Protein Cell.

[18] Cederroth CR (2019). Medicine in the fourth dimension. Cell Metab..

[19] Schroeder AM, Colwell CS (2013). How to fix a broken clock. Trends Pharm. Sci..

[20] Kohsaka A (2007). High-fat diet disrupts behavioral and molecular circadian rhythms in mice. Cell Metab..

[21] Acosta-Rodriguez VA, Rijo-Ferreira F, Green CB, Takahashi JS (2021). Importance of circadian timing for aging and longevity. Nat. Commun..

[22] Chen Z (2012). Identification of diverse modulators of central and peripheral circadian clocks by high-throughput chemical screening. Proc. Natl Acad. Sci. USA.

[23] Hirota T (2008). A chemical biology approach reveals period shortening of the mammalian circadian clock by specific inhibition of GSK-3beta. Proc. Natl Acad. Sci. USA.

[24] Ju, D. et al. Chemical perturbations reveal that RUVBL2 regulates the circadian phase in mammals. *Sci. Transl. Med.* **12**, 10.1126/scitranslmed.aba0769 (2020). 10.1126/scitranslmed.aba0769 32376767

[25] Solt LA (2012). Regulation of circadian behaviour and metabolism by synthetic REV-ERB agonists. Nature.

[26] Banerjee S (2014). Pharmacological targeting of the mammalian clock regulates sleep architecture and emotional behaviour. Nat. Commun..

[27] He B (2016). The small molecule nobiletin targets the molecular oscillator to enhance circadian rhythms and protect against metabolic syndrome. Cell Metab..

[28] Nohara K (2019). Nobiletin fortifies mitochondrial respiration in skeletal muscle to promote healthy aging against metabolic challenge. Nat. Commun..

[29] Hirota T (2012). Identification of small molecule activators of cryptochrome. Science.

[30] Sulli G (2018). Pharmacological activation of REV-ERBs is lethal in cancer and oncogene-induced senescence. Nature.

[31] Humphries PS (2016). Carbazole-containing sulfonamides and sulfamides: discovery of cryptochrome modulators as antidiabetic agents. Bioorg. Med Chem. Lett..

[32] Chen Z, Yoo SH, Takahashi JS (2013). Small molecule modifiers of circadian clocks. Cell Mol. Life Sci..

[33] Chen Z, Yoo SH, Takahashi JS (2018). Development and therapeutic potential of small-molecule modulators of circadian systems. Annu Rev. Pharm. Toxicol..

[34] Straif K (2007). Carcinogenicity of shift-work, painting, and fire-fighting. Lancet Oncol..

[35] Jakubowicz D (2015). High-energy breakfast with low-energy dinner decreases overall daily hyperglycaemia in type 2 diabetic patients: a randomised clinical trial. Diabetologia.

[36] Scheer FA, Hilton MF, Mantzoros CS, Shea SA (2009). Adverse metabolic and cardiovascular consequences of circadian misalignment. Proc. Natl Acad. Sci. USA.

[37] Schneider JW (2008). Small-molecule activation of neuronal cell fate. Nat. Chem. Biol..

[38] Yoo SH (2004). PERIOD2::LUCIFERASE real-time reporting of circadian dynamics reveals persistent circadian oscillations in mouse peripheral tissues. Proc. Natl Acad. Sci. USA.

[39] Di Francesco A, Di Germanio C, Bernier M, de Cabo R (2018). A time to fast. Science.

[40] Kinouchi K (2018). Fasting imparts a switch to alternative daily pathways in liver and muscle. Cell Rep..

[41] Zhu WZ (2003). Linkage of beta1-adrenergic stimulation to apoptotic heart cell death through protein kinase A-independent activation of Ca2+/calmodulin kinase II. J. Clin. Invest.

[42] Bhattacharyya, M., Karandur, D. & Kuriyan, J. Structural insights into the regulation of Ca(2+)/calmodulin-dependent protein kinase II (CaMKII). *Cold Spring Harb. Perspect. Biol.* **12**, 10.1101/cshperspect.a035147 (2020). 10.1101/cshperspect.a035147 PMC7263085 31653643

[43] Kabakov AY, Lisman JE (2015). Catalytically dead alphaCaMKII K42M mutant acts as a dominant negative in the control of synaptic strength. PLoS One.

[44] Blair RE, Churn SB, Sombati S, Lou JK, DeLorenzo RJ (1999). Long-lasting decrease in neuronal Ca2+/calmodulin-dependent protein kinase II activity in a hippocampal neuronal culture model of spontaneous recurrent seizures. Brain Res..

[45] Li X (2015). Small-molecule-driven direct reprogramming of mouse fibroblasts into functional neurons. Cell Stem Cell.

[46] Bettio LE (2016). ISX-9 can potentiate cell proliferation and neuronal commitment in the rat dentate gyrus. Neuroscience.

[47] Galinato MH (2018). A synthetic small-molecule Isoxazole-9 protects against methamphetamine relapse. Mol. Psychiatry.

[48] Staples MC (2021). Isoxazole-9 reduces enhanced fear responses and retrieval in ethanol-dependent male rats. J. Neurosci. Res..

[49] Consortium, H. D. i. (2017). Developmental alterations in Huntington’s disease neural cells and pharmacological rescue in cells and mice. Nat. Neurosci..

[50] Pujol, J. B. et al. Isx9 regulates calbindin D28K expression in pancreatic beta cells and promotes beta cell survival and function. *Int. J. Mol. Sc.* **19**10.3390/ijms19092542 (2018). 10.3390/ijms19092542 PMC6165483 30150605

[51] Yoshitane H, Fukada Y (2021). Circadian phosphorylation of CLOCK and BMAL1. Methods Mol. Biol..

[52] Tamaru T (2009). CK2alpha phosphorylates BMAL1 to regulate the mammalian clock. Nat. Struct. Mol. Biol..

[53] Sahar S, Zocchi L, Kinoshita C, Borrelli E, Sassone-Corsi P (2010). Regulation of BMAL1 protein stability and circadian function by GSK3beta-mediated phosphorylation. PLoS ONE.

[54] Sanada K, Okano T, Fukada Y (2002). Mitogen-activated protein kinase phosphorylates and negatively regulates basic helix-loop-helix-PAS transcription factor BMAL1. J. Biol. Chem..

[55] Colwell CS (2000). Circadian modulation of calcium levels in cells in the suprachiasmatic nucleus. Eur. J. Neurosci..

[56] Ikeda M (2003). Circadian dynamics of cytosolic and nuclear Ca2+ in single suprachiasmatic nucleus neurons. Neuron.

[57] Nitabach MN (2005). Circadian rhythms: clock coordination. Nature.

[58] Lundkvist GB, Kwak Y, Davis EK, Tei H, Block GD (2005). A calcium flux is required for circadian rhythm generation in mammalian pacemaker neurons. J. Neurosci..

[59] Enoki R (2012). Topological specificity and hierarchical network of the circadian calcium rhythm in the suprachiasmatic nucleus. Proc. Natl Acad. Sci. USA.

[60] Pennartz CM, de Jeu MT, Bos NP, Schaap J, Geurtsen AM (2002). Diurnal modulation of pacemaker potentials and calcium current in the mammalian circadian clock. Nature.

[61] Kon N (2014). CaMKII is essential for the cellular clock and coupling between morning and evening behavioral rhythms. Genes Dev..

[62] Storch KF (2007). Intrinsic circadian clock of the mammalian retina: importance for retinal processing of visual information. Cell.

[63] Savelyev, S. A., Larsson, K. C., Johansson, A. S. & Lundkvist, G. B. Slice preparation, organotypic tissue culturing and luciferase recording of clock gene activity in the suprachiasmatic nucleus. *J. Vis. Exp.*10.3791/2439 (2011). 10.3791/2439 PMC3197397 21372784

[64] Alexandre C (2017). Decreased alertness due to sleep loss increases pain sensitivity in mice. Nat. Med..

[65] Laposky A (2005). Deletion of the mammalian circadian clock gene BMAL1/Mop3 alters baseline sleep architecture and the response to sleep deprivation. Sleep.

