# Supplementary material for: ISX-9 potentiates CaMKIIδ-mediated BMAL1 activation to enhance circadian amplitude
Source: Commun Biol. 2022 Jul 28;5:750. doi: 10.1038/s42003-022-03725-x (PMC9334596; doi:10.1038/s42003-022-03725-x)
Supplement: Supplementary file 1 — Supplementary Information [file 42003_2022_3725_MOESM1_ESM.pdf]

ISX-9 potentiates CaMKIIδ-mediated BMAL1 activation to enhance circadian amplitude

Supplementary Figures

Supplementary Figure 1. Small molecule examples revealed with PER2::LUC amplitude-decreasing or period-changing effect.

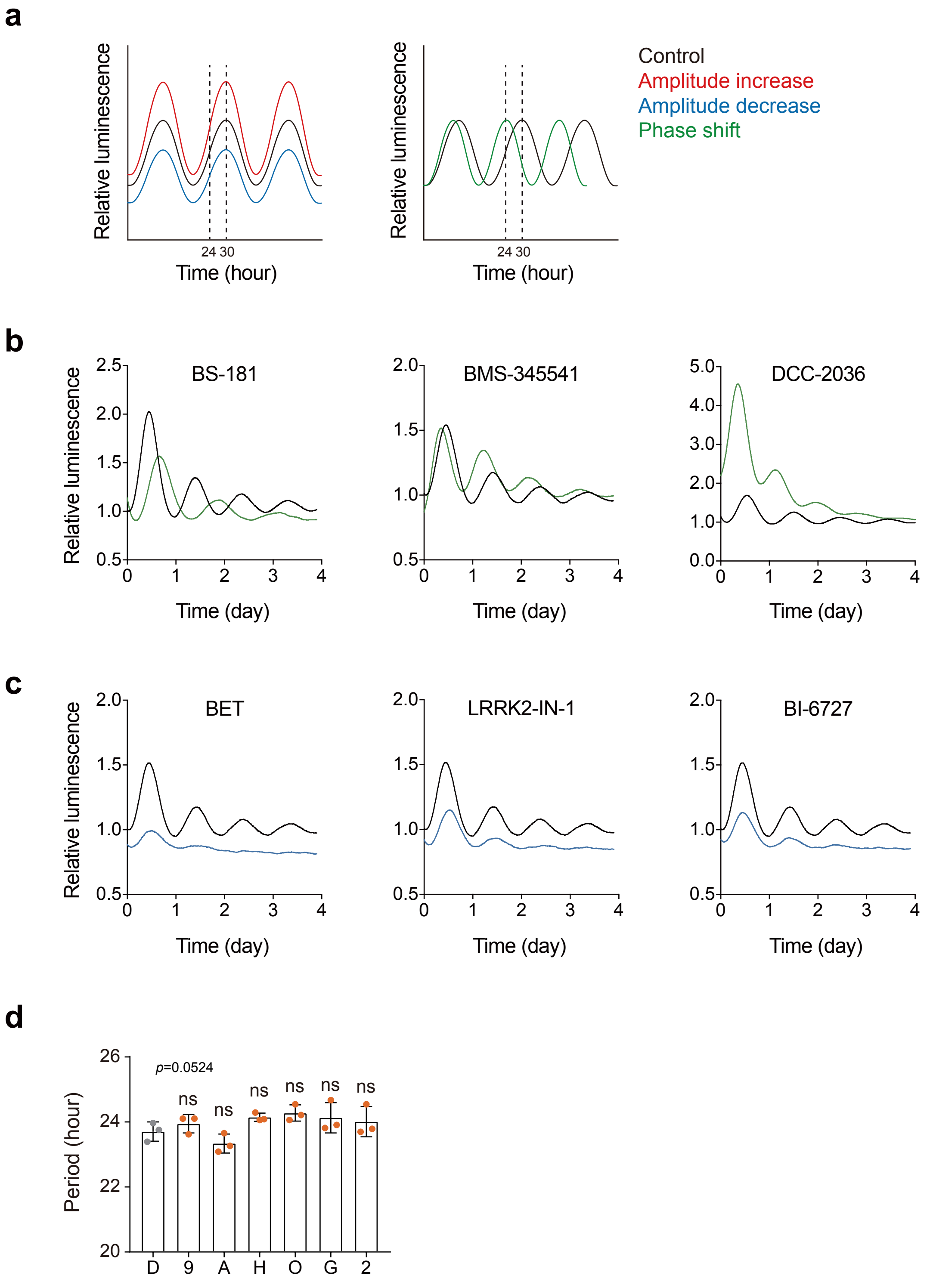

a, Schematic diagrams of compound effect on amplitude or period, distinguished by luminescence fluctuations between time points 24-hr and 30-hr. **b, c,** Real-time bioluminescence recordings of synchronized mPer2Luc MEF cells with DMSO (black curve) and indicated small molecules which period alteration (**b,** green curve) or decreased circadian amplitude (**c,** blue curve). **d,** Period of indicated small molecules associated with Fig. 1d. 9: ISX-9; A: Atrasentan; H: HhAntag; O: OSI-930; G: GW6064 and 2: 2-NP. One-way ANOVA with Bonferroni correction for multiple comparison was performed, (ns, not significant, ## $p < 0.01$, ### $p < 0.001$) and data are shown as mean ± SD.

Supplementary Figure 2. ISX-9 enhanced circadian amplitude in a BMAL1-dependent manner.

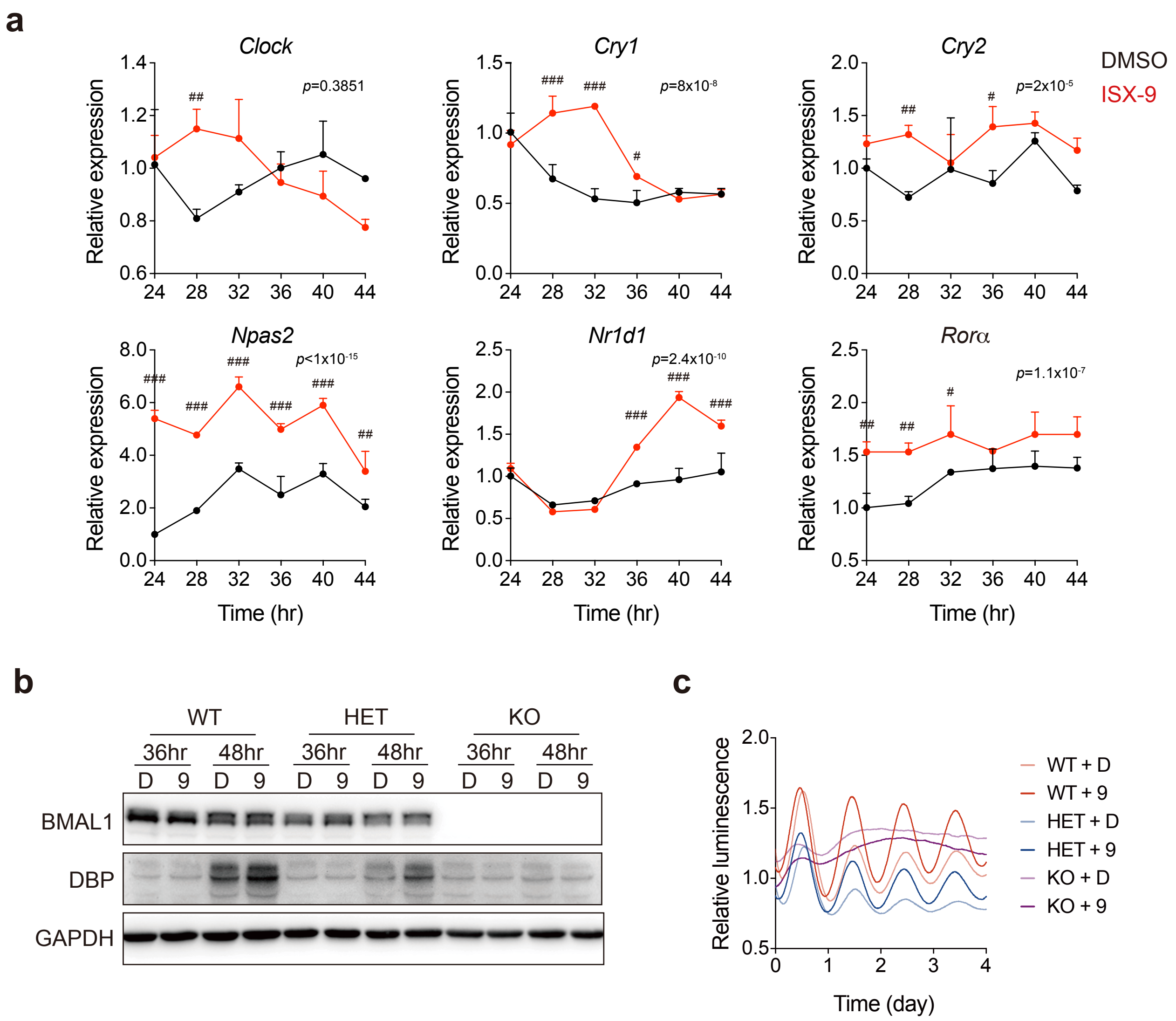

a, Quantitative PCR analyses of clock gene expressions in MEFs treated with vehicle (black) or ISX-9 (red) at different time points. Two-way ANOVA with Bonferroni correction for multiple comparison was performed, with p value labelled on top. Significances between DMSO and ISX-9-treated cells in each time point were shown (# $p < 0.05$, ## $p < 0.01$, ### $p < 0.001$), and data are shown as mean ± SD. **b,** Immunoblots of BMAL1 and DBP from Bmal1wild-type (WT), heterozygote (HET) or knock-out (KO) MEF cells treated with DMSO (D) or 10 μM ISX-9 (9), then harvested at 36-hr or 48-hr time point post treatment. **c,** Real-time bioluminescence recording of synchronized PER2::LUC from Bmal1 wild-type (WT), heterozygote (HET) or knock-out (KO) MEF cells treated with DMSO (D) or 10 μM ISX-9 (9).

Supplementary Figure 3. ISX-9 rejuvenated metabolic oscillations in 18-month-old mice.

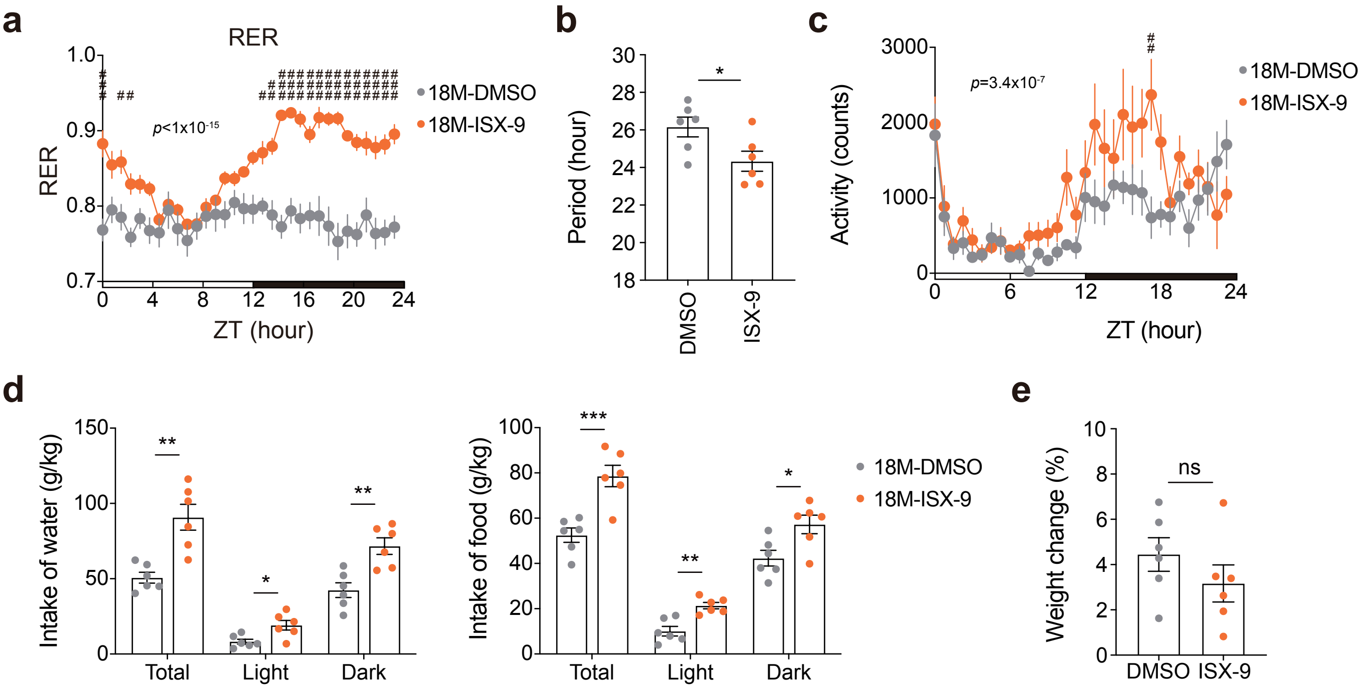

a, Respiratory exchange ratio (RER) of male 18-month-old mice with DMSO (grey curve, n=6) or ISX-9 (orange curve, n=6). **b,** Period of RER fluctuation from the metabolic cage data, processed with software ClockLab. **c,** Diurnal activity in the 24-hour cycle, **d,** daily water intake and food consumption, and **e,** body weight change in mouse cohorts with or without ISX-9 treatment. **a and c,** All data represented as mean ± SEM. Two-way ANOVA with Bonferroni correction for multiple comparison was performed, with *p* value labelled on top. Significances between DMSO and ISX-9-treated mouse cohorts in each time point were shown (#$p < 0.05$, ## $p < 0.01$, ### $p < 0.001$). **b, d and e,** Unpaired Student's t test was used (ns, not significant, * $p < 0.05$, ** $p < 0.01$), and data are shown as mean ± SEM.

Supplementary Figure 4. Diurnal EEG and physical activities in juvenile versus middle-aged mouse cohorts.

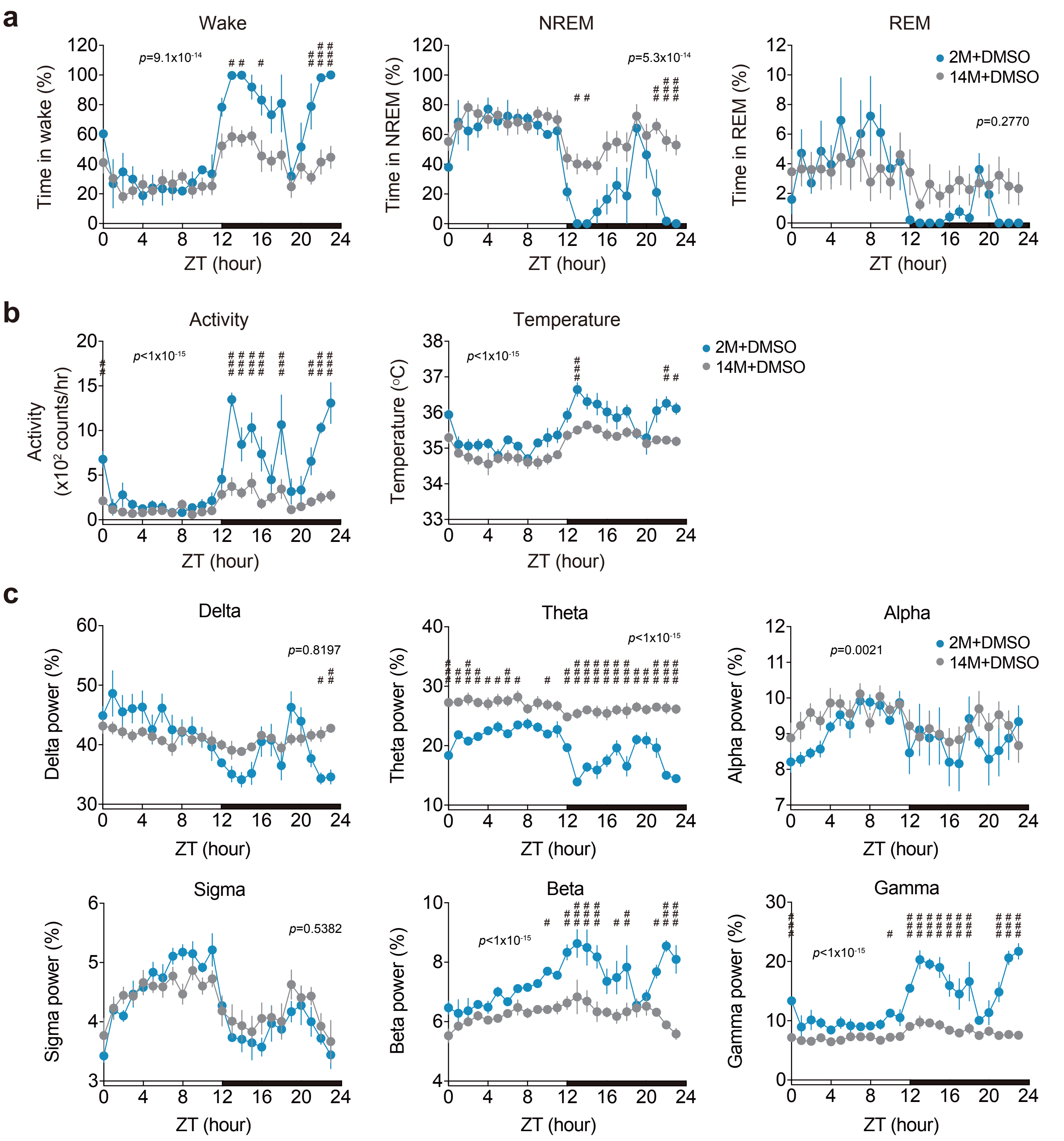

a, Percentage of time in wakefulness, NREM sleep and REM sleep of male 2-month-old (blue, n=4) versus male 14-month-old mice (grey, n=8). Bottom indicates the light-on period (white section, 07:00-19:00) and light-off period (black section, 19:00-07:00), respectively. **b,** Diurnal activity and body temperature changes in the 24-hour cycle of male 2-month-old (blue, n=4) versus male 14-month-old mice (grey, n=8). **c,** EEG power analysis in male 2-month-old (blue, n=4) versus male 14-month-old mice (grey, n=8). The proportion of absolute powers during a circadian day for delta (0.5-4 Hz), theta (4-8 Hz), alpha (8-12 Hz), sigma (12-16 Hz), beta (16-32 Hz) and gamma (>32 Hz) were shown. Two-way ANOVA with Bonferroni correction for multiple comparison was performed, with *p* value labelled on top. Significances between DMSO and ISX-9-treated mouse cohorts in each time point were shown (#*p* < 0.05, ##*p* < 0.01, ### *p* < 0.001), and data are shown as mean ± SEM.

Supplementary Figure 5. Isoproterenol moderately enhanced PER2::LUC amplitudes without altering circadian period.

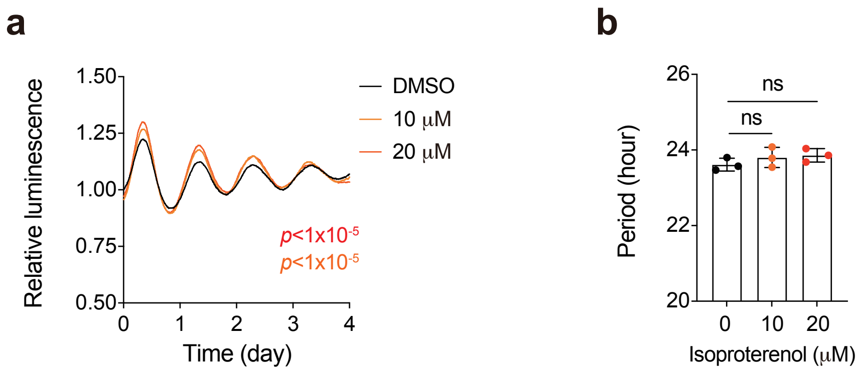

a, Dose-dependent effects of Isoproterenol in the *mPer2*^{Luc} MEFs. Two-way ANOVA with Bonferroni correction for multiple comparison was performed. **b,** Period of PER2::LUC post Isoproterenol treatment, data associated with a. Unpaired Student's t test was used (ns, not significant), and data are shown as mean ± SEM.

Supplementary Figure 6. Single-molecule RNA fluorescence in situ hybridization of Camk2 isoforms in wild-type mouse brain.

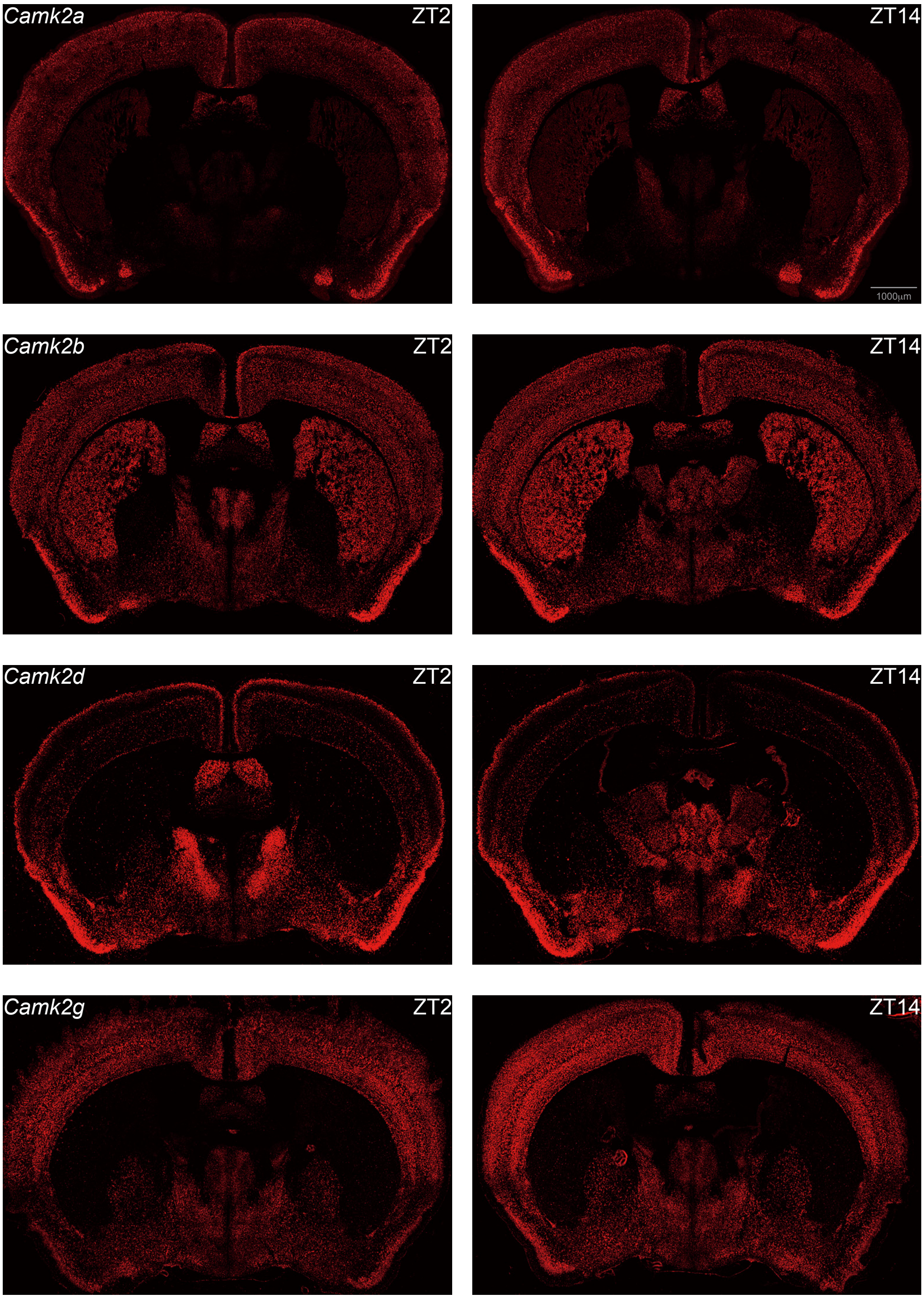

Single-molecule RNA fluorescence in situ hybridization of *CamK2a*, *b*, *d* and *g* in coronal sections from male 2-month-old wild-type mice at ZT2 or ZT14. *Camk2d* showed higher expression in sub-cortical regions and the hypothalamus. *Camk2a* and *Camk2b* showed distinct, higher expression in the striatum and cortex. Scale bar, 1000 μm.

Supplementary Figure 7. ISX-9 re-activated *Camk2d* expression in the SCN of aged mice.

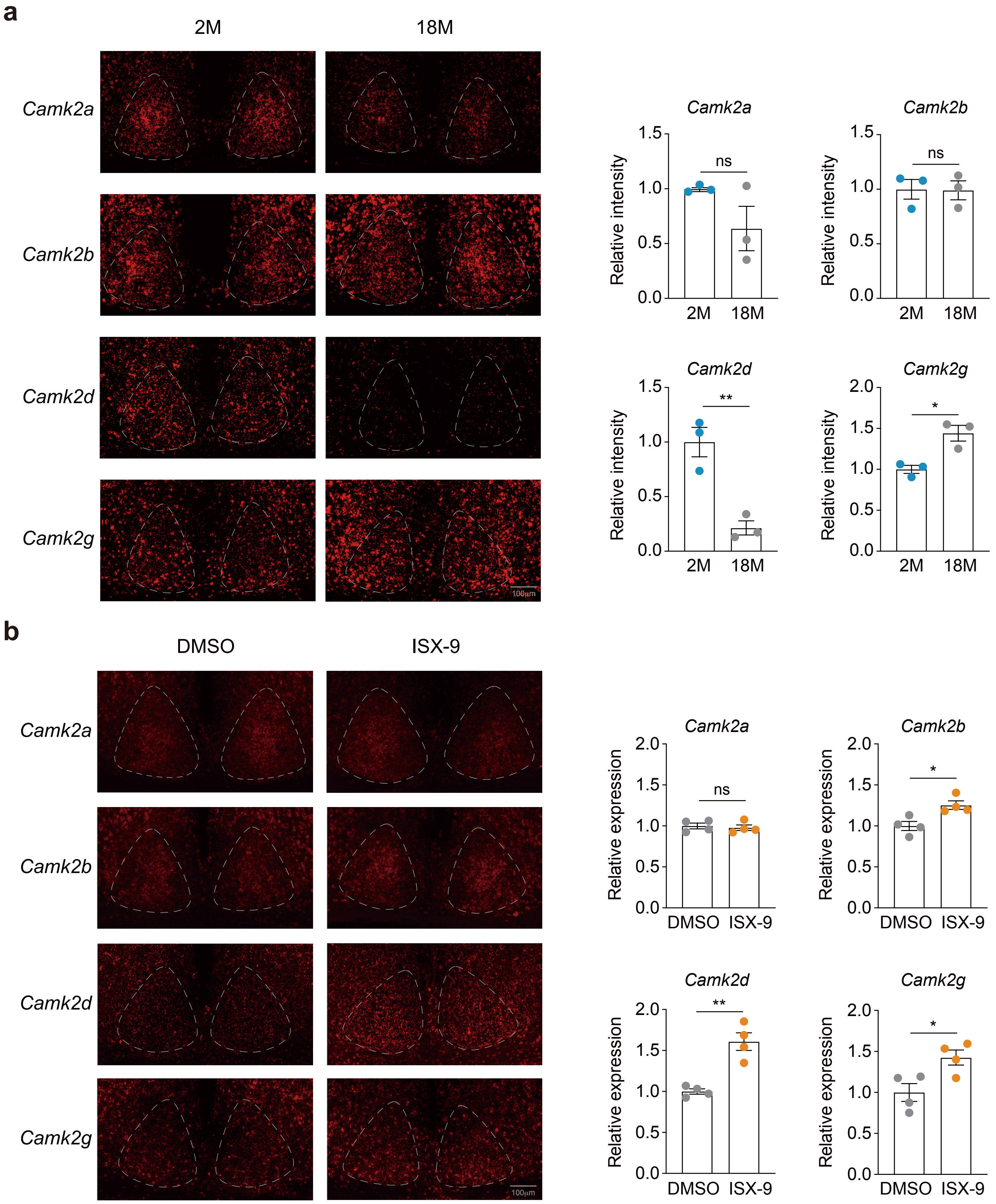

a, smFISH detection of *CamK2a*, *b*, *d* and *g* in the SCN of male 2-month-old (n=3) and male 18-month-old mice (n=3) in ZT10. Expression levels were quantified from the SCN region, then set the intensity of each *Camk2* isoforms in 2-month-old mice as 1. **b,** smFISH detection of *CamK2a*, *b*, *d* and *g* in the SCN of male 16-month-old mice treated with DMSO or ISX-9. Expression levels were quantified and the average intensity from DMSO-treated group was set as 1 (n=4/group). Unpaired Student's t test was used (ns, not significant, * $p < 0.05$, ** $p < 0.01$), and data are shown as mean ± SEM.

Supplementary Figure 8. *Camk2d* is a clock-controlled gene that responds to CLOCK/BMAL1 activation.

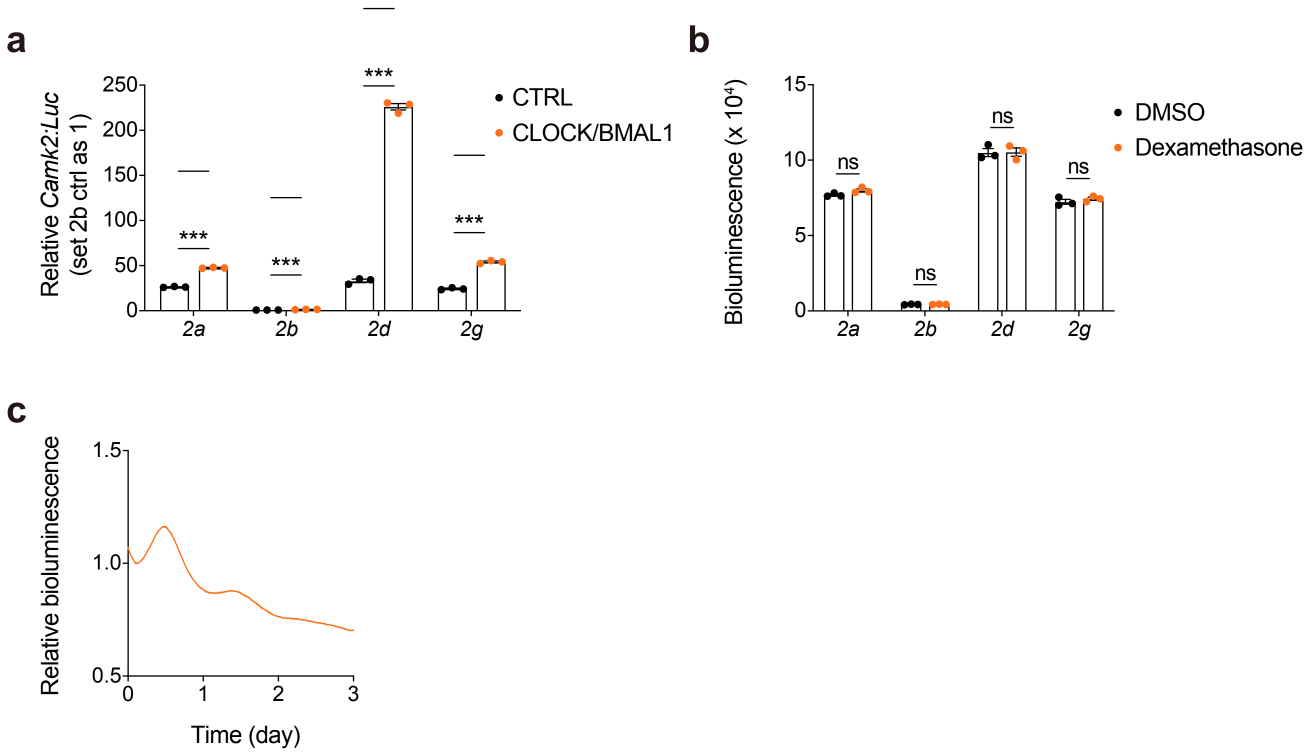

a, *Camk2*:Luc bioluminescence assay in N2a cells treated with or without CLOCK/BMAL1 co-expression. **b,** Bioluminescence assay in N2a cells that were transfected with *Camk2*:Luc reporter plasmids. Cells were treated with DMSO or 100 nM dexamethasone for 15 minutes, then cultured in DMEM for 24 hours before the cell lyaste preparation for luciferase measurement. **c,** Real-time bioluminescence recording of transfected *Camk2d*:Luc expression in MEF cells. **a and b,** Unpaired Student's t test was used (ns, not significant, *** $p < 0.001$), and data are shown as mean ± SD.

Supplementary Figure 9. BMAL1 3A and CaMKIIδ K43M mutants affected CLOCK/BMAL1 activity in regulating *Dbp*:Luc expression.

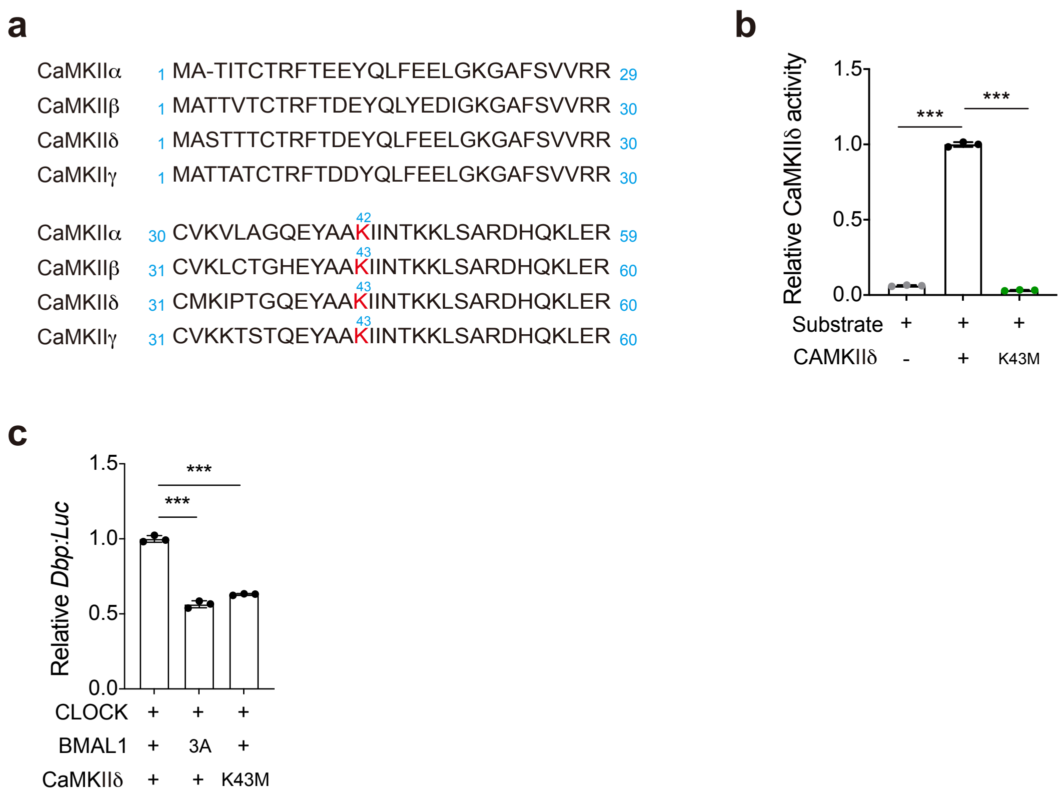

a, Amino acid sequence alignment of CaMKII α, β, δ and γ. K42 (CaMKII α) and K43 (other CaMKIIs) were marked in red. **b,** In vitro kinase assay of purified wild-type CaMKII δ and K43M mutant using autocamtide-2 peptide (KKALRRQETVDAL) as the substrate. **c,** *Dbp*:Luc bioluminescence assay with wild-type CLOCK, BMAL1 and CaMKII δ co-expression, or replaced with mutant BMAL1-S513A/S515A/S516A or CaMKII δ K43M co-expression. Unpaired Student's t test was used (****p* < 0.001), and data are shown as mean ± SD.

Original Blot Scans

Fig 2e

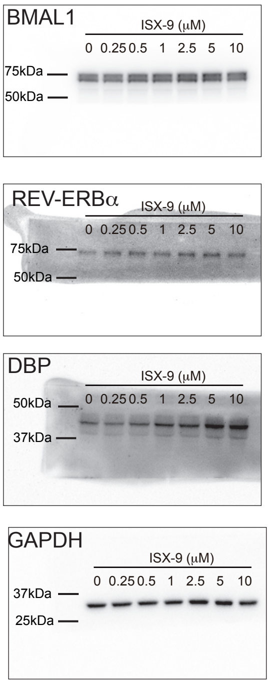

Fig S2b

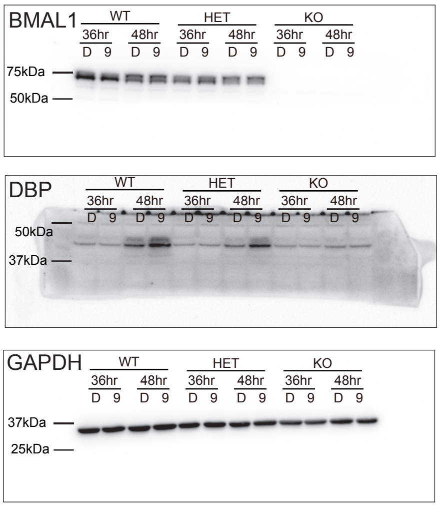

Fig 5d

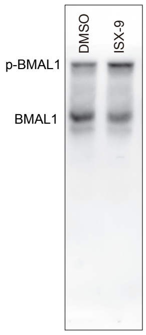

Supplementary Table 1. Primers for plasmid construction

Bmal1	
Forward (5'-3')	ATATGCGGCCGCGCGGACCAGAGAATGGAC
Reverse (5'-3')	ACCTTCACGCGTCTACAGCGGCCATGGCAAGTCACTA
Bmal1 S513A	
Forward (5'-3')	CCACAGGATAAGAGGGTCAGCGCCTTCCAGCTGTGGCTCCA
Reverse (5'-3')	TGACCCTCTTATCCTGTGGAT
Bmal1 S515A	
Forward (5'-3')	CAGGATAAGAGGGTCATCGCCTGCAAGCTGTGGCTCCAGCCCG
Reverse (5'-3')	TGACCCTCTTATCCTGTGGAT
Bmal1 S516A	
Forward (5'-3')	AAGAGGGTCATCGCCTTCCGCATGTGGCTCCAGCCCGCTGA
Reverse (5'-3')	GGAAGGCGATGACCCTCTTATCC
Bmal1 S519A	
Forward (5'-3')	CATCGCCTTCCAGCTGTGGCGCCAGCCCGCTGAACATCACA
Reverse (5'-3')	GCCACAGCTGGAAGGCGATG
Bmal1 S513A/S515A/S516A	
Forward (5'-3')	AATCCACAGGATAAGAGGGTCAGCGCCTGCCGCCTGTGGCTCCAGCCCGCTGAAC
Reverse (5'-3')	TGACCCTCTTATCCTGTGGAT
Camk2a	
Forward (5'-3')	GATGACGACGATAAGTGCGGCCGCGCTACCATCACCTGCACC
Reverse (5'-3')	TCCCTGGTACCTTCACGCGTTCACTGTAGCGGTGCGGCAG
Camk2b	
Forward (5'-3')	GGATGACGACGATAAGTGCGGCCGCGCCACCACGGTGACCTGCA
Reverse (5'-3')	CCCTGGTACCTTCACGCGTTCACTGCAGCGGGGCCA
Camk2d	
Forward (5'-3')	GATGACGACGATAAGTGCGGCCGCGCTTCGACCACCACCTGCAC
Reverse (5'-3')	CCCTGGTACCTTCACGCGTTTAGTTGATGGGTACTGTGGG
Camk2g	
Forward (5'-3')	GATGACGACGATAAGTGCGGCCGCGCCACCACCGCCACCTGCAC
Reverse (5'-3')	TCCCTGGTACCTTCACGCGTTCACTGTAGCGGTGCGGCAG
Camk2d K43M	
Forward (5'-3')	TGGACAAGAGTATGCTGCCATGATTATCAACACCAAAAAG

Reverse (5'-3')	GGCAGCATACTCTTGTCCA
Dbp promoter	
Forward (5'-3')	TAGACGCGTGCCCACTTCTTTCTGCCTTA
Reverse (5'-3')	GCGAGATCTTGCAGAAAGGTGCAACTCAAA
Rev-erbα promoter	
Forward (5'-3')	CTTACGCGTCTAGTCACCACTAACCTCAG
Reverse (5'-3')	GATCTCGAGGCAACCAGGAAGTAAGTAGG
Camk2a promoter	
Forward (5'-3')	CTTACGCGTTACATCTACACCACCCGCCC
Reverse (5'-3')	CGCAGATCTGCTTTAGGCTGGGAACTAGGG
Camk2b promoter	
Forward (5'-3')	CTTACGCGTATCCAGAAGATCGCCGAACAG
Reverse (5'-3')	GCGAGATCTGCTCGCTCTGTCCCC
Camk2d promoter	
Forward (5'-3')	CTTACGCGTCCACCACTTACACCTTAACCCA
Reverse (5'-3')	CGCAGATCTCACGGAGAGCTCAACGCA
Camk2g promoter	
Forward (5'-3')	CTTACGCGTCCAGACATCCGAGAGCTAGGTA
Reverse (5'-3')	CGCAGATCTATACTGGCGGGCGGACGCGG

Supplementary Table 2. Primers for real-time quantitative PCR

Genes	Forward primers (5'-3')	Reverse primers (5'-3')
Bmal1	CTTGCAAGCACCTTCCTTCC	GGGTCATCTTTGTCTGTGTC
Clock	CTTCCTGGTAACGCGAGAAAG	GTCGAATCTCACTAGCATCTGAC
Cry1	TTCCCTCCCTTGAAGCTCTC	GAAGCAAAAATCGCCACCTG
Cry2	CACTGGTTCCGCAAAGGACTA	CCACGGGTCGAGGATGTAGA
Dbp	CTGGCCCGAGTCTTTTTGC	CCAGGTCCACGTATTCCACG
Npas2	CGTCGGGACCAGTTCAATGTT	AGCACGGTGGTTTTGTCCAT
Nr1d1	ACTTCCCACCATCACCTACTG	GGGGAGCTATCATCACTGAGA
Per2	GCGAAGCGCTTATTCCAGAG	AGTCTGAAGGCATCATCAGG
Rora	ACGCCCACCTACAACATCTC	ACATATGGGTTCGGGTTTGA
Rpl19	AAGCCTGTGACTGTCCATTC	CTTCTTGGATTCCCGG
