# Supplementary material for: ISX-9 potentiates CaMKIIδ-mediated BMAL1 activation to enhance circadian amplitude
Source: Commun Biol. 2022 Jul 28;5:750. doi: 10.1038/s42003-022-03725-x (PMC9334596; doi:10.1038/s42003-022-03725-x)
Supplement: Supplementary file 2 — Description of Additional Supplementary Files [file 42003_2022_3725_MOESM2_ESM.pdf]

Description of Additional Supplementary Files

File name: Supplementary Data 1

Description: Screen results that are associated with Fig. 1b and Fig 1c.

File name: Supplementary Data 2

Description: Source data for graphs and charts in this study.
