# Supplementary material for: ISX-9 potentiates CaMKIIδ-mediated BMAL1 activation to enhance circadian amplitude
Source: Commun Biol. 2022 Jul 28;5:750. doi: 10.1038/s42003-022-03725-x (PMC9334596; doi:10.1038/s42003-022-03725-x)
Supplement: Supplementary file 5 — Reporting Summary [file 42003_2022_3725_MOESM5_ESM.pdf]

nature portfolio

Corresponding author(s): Yun-Chi Tang and Hung-Chun Chang

Last updated by author(s): Jun 28, 2022

Reporting Summary

Nature Portfolio wishes to improve the reproducibility of the work that we publish. This form provides structure for consistency and transparency in reporting. For further information on Nature Portfolio policies, see our Editorial Policies and the Editorial Policy Checklist.

Statistics

For all statistical analyses, confirm that the following items are present in the figure legend, table legend, main text, or Methods section.

n/a	Confirmed	
☐	☒	The exact sample size (n) for each experimental group/condition, given as a discrete number and unit of measurement
☐	☒	A statement on whether measurements were taken from distinct samples or whether the same sample was measured repeatedly
☐	☒	The statistical test(s) used AND whether they are one- or two-sided<br>*Only common tests should be described solely by name; describe more complex techniques in the Methods section.*
☒	☐	A description of all covariates tested
☐	☒	A description of any assumptions or corrections, such as tests of normality and adjustment for multiple comparisons
☐	☒	A full description of the statistical parameters including central tendency (e.g. means) or other basic estimates (e.g. regression coefficient) AND variation (e.g. standard deviation) or associated estimates of uncertainty (e.g. confidence intervals)
☐	☒	For null hypothesis testing, the test statistic (e.g. F, t, r) with confidence intervals, effect sizes, degrees of freedom and P value noted<br>*Give P values as exact values whenever suitable.*
☒	☐	For Bayesian analysis, information on the choice of priors and Markov chain Monte Carlo settings
☒	☐	For hierarchical and complex designs, identification of the appropriate level for tests and full reporting of outcomes
☒	☐	Estimates of effect sizes (e.g. Cohen's d, Pearson's r), indicating how they were calculated

Our web collection on statistics for biologists contains articles on many of the points above.

Software and code

Policy information about availability of computer code

Data collection	ClockLab, Image J, Ponemah
Data analysis	CLAX (Columbus Instruments), NeuroScore 3.2.0 (DSI),GraphPad Prism 7.0, ClockLab, MATLAB

For manuscripts utilizing custom algorithms or software that are central to the research but not yet described in published literature, software must be made available to editors and reviewers. We strongly encourage code deposition in a community repository (e.g. GitHub). See the Nature Portfolio guidelines for submitting code & software for further information.

Data

Policy information about availability of data

All manuscripts must include a data availability statement. This statement should provide the following information, where applicable:

- Accession codes, unique identifiers, or web links for publicly available datasets
- A description of any restrictions on data availability
- For clinical datasets or third party data, please ensure that the statement adheres to our policy

The datasets generated and analyzed during the current study are available from the corresponding authors upon request.

Field-specific reporting

Please select the one below that is the best fit for your research. If you are not sure, read the appropriate sections before making your selection.

☒ Life sciences ☐ Behavioural & social sciences ☐ Ecological, evolutionary & environmental sciences

For a reference copy of the document with all sections, see nature.com/documents/nr-reporting-summary-flat.pdf

Life sciences study design

All studies must disclose on these points even when the disclosure is negative.

Sample size	For animal studies, the sample sizes were determined based on previous experiments using similar methodologies (PMID: 27238018, 27076076, 16171284, 28481358) We randomly assigned age-matched mice to drug versus vehicle treatments to minimize any potential bias.
Data exclusions	No data was excluded.
Replication	All in vitro and ex vivo results were reproduced in independent experiments for at least three times as indicated in the associated legends, except for the primary screen of small molecules. All animal experiments were performed for at least twice with independent cohort of mice.
Randomization	No randomization was applied for the in vitro cell culture experiments. For animal experiments, we assigned age-matched mice randomly to groups administrated with drug or vehicle.
Blinding	Animal treatments were not done blind. However, standard randomization procedures were strictly followed with proper controls included in all experiments. Biological samples, and physiological data were collected and analyzed under the same conditions.

Reporting for specific materials, systems and methods

We require information from authors about some types of materials, experimental systems and methods used in many studies. Here, indicate whether each material, system or method listed is relevant to your study. If you are not sure if a list item applies to your research, read the appropriate section before selecting a response.

Materials & experimental systems

n/a	Involved in the study	
☐	☒	Antibodies
☐	☒	Eukaryotic cell lines
☒	☐	Palaeontology and archaeology
☐	☒	Animals and other organisms
☒	☐	Human research participants
☒	☐	Clinical data
☒	☐	Dual use research of concern

Methods

n/a	Involved in the study	
☒	☐	ChIP-seq
☒	☐	Flow cytometry
☒	☐	MRI-based neuroimaging

Antibodies

Antibodies used	Anti-BMAL1 (Cell Signaling Technology, 14020), Anti-PER2 (Acris,per21-A), Anti-DBP (Proteintech, 12662-1-AP), Anti-REV-ERBa (Cell Signaling Technology, 13418), Anti-GAPDH (Proteintech, HRP-60004), Anti-FLAG (Sigma-Aldrich, F1804), HRP-conjugated goat anti-rabbit secondary antibody (Bio-Rad, 1706515), HRP-conjugated goat anti-mouse secondary antibody (Bio-Rad, 1706516)
Validation	Only commercially available antibodies were used. All antibodies used have been tested and validated by the suppliers and also by other researchers and ourselves.

Eukaryotic cell lines

Policy information about cell lines

Cell line source(s)	HEK293T, NIH-3T3 and N2a cells used in this study are commercial and were obtained from ATCC.
Authentication	No authentication was used.
Mycoplasma contamination	All cells used in this study were mycoplasma negative.
Commonly misidentified lines (See ICLAC register)	No commonly misidentified cell lines were used.

Animals and other organisms

Policy information about studies involving animals; ARRIVE guidelines recommended for reporting animal research

Laboratory animals	mPer2Luc (006852) and Bmal1-floxed conditional strain (007668) were obtained from the Jackson Laboratory and maintained in C57BL/6J background. For drug treatment tests, male C57BL/6 mice at 2, 3, 14, 16 and 18 months of ages were raised and maintained in our colony at Institute of Neuroscience, CAS.
Wild animals	No wild animal was used.
Field-collected samples	No field-collected sample was used.
Ethics oversight	All animal procedures were reviewed and approved by the institutional Biomedical Research Ethics Committee, Shanghai Institute of Nutrition and Health and Center for Excellence in Brain Science and Intelligence Technology, Chinese Academy of Sciences

Note that full information on the approval of the study protocol must also be provided in the manuscript.
